# Error analysis for discretizations of parabolic problems using continuous finite elements in time and mixed finite elements in space

**DOI:** 10.1007/s00211-017-0894-6

**Published:** 2017-06-20

**Authors:** Markus Bause, Florin A. Radu, Uwe Köcher

**Affiliations:** 1 Faculty of Mechanical Engineering, Helmut Schmidt University, Holstenhofweg 85, 220433 Hamburg, Germany; 2 Department of Mathematics, University of Bergen, Allégaten 41, 50520 Bergen, Norway

**Keywords:** 65M12, 65M60, 76S05

## Abstract

Variational time discretization schemes are getting of increasing importance for the accurate numerical approximation of transient phenomena. The applicability and value of mixed finite element methods in space for simulating transport processes have been demonstrated in a wide class of works. We consider a family of continuous Galerkin–Petrov time discretization schemes that is combined with a mixed finite element approximation of the spatial variables. The existence and uniqueness of the semidiscrete approximation and of the fully discrete solution are established. For this, the Banach–Nečas–Babuška theorem is applied in a non-standard way. Error estimates with explicit rates of convergence are proved for the scalar and vector-valued variable. An optimal order estimate in space and time is proved by duality techniques for the scalar variable. The convergence rates are analyzed and illustrated by numerical experiments, also on stochastically perturbed meshes.

## Introduction

Numerical simulations of time dependent single and multiphase phase flow and multicomponent transport processes in complex and porous media with strong heterogeneities and anisotropies are desirable in several fields of natural sciences and civil engineering as well as in a large number of branches of technology; cf. e.g., [22, 29]. Typically, the discretization in space involves a significant set of complexities and challenges. MFEM (cf. [17, 21]) have proved their potential and capability to approximate solutions with high accuracy and physical consistency; cf. e.g., [13, 19]. So far, the temporal approximation of flows and transport phenomena in porous media have received relatively little interest (cf. e.g., [5, 18, 27, 42–44, 49] and the references therein) and have been limited to traditional non-adaptive first and second order methods, even if strong chemical reactions with high temporal variations in profiles are present. Rigorous studies of higher order time discretizations are still missing. The low-order implicit time discretization is of particular concern with respect to numerical diffusion for smooth solutions of transport problems (cf. [45] for a study on numerical diffusion for different temporal and spatial discretizations of a transport equation).

The Galerkin method is a well-recognised approach to solve time dependent problems; cf. e.g., [6, 48]. However, until now it has rarely been used in practice for discretizing the time variable in approximations of initial-boundary value problems. Since recently, variational time discretization schemes based on continuous or discontinuous finite element techniques have been developed to the point that they can be put into use (cf. [30, 31]) and demonstrate their significant advantages. Higher order methods are naturally embedded in these schemes and the uniform variational approach simplifies stability and error analyses. Further, goal-oriented error control [9] based on the dual weighted residual approach relies on variational space-time formulations and the concepts of adaptive finite element techniques for changing the polynomial degree as well as the length of the time intervals become applicable. Variational time discretization schemes that are combined with continuous or discontinuous finite element methods for the spatial variables are studied for flow and parabolic problems in, for instance [1–4, 10, 15, 30–32, 38, 47] and for wave problems in, for instance [7, 36, 37]. In these works algebraic formulations of the variational time discretizations are developed [4, 30, 31, 36, 37, 47], preconditioning techniques for the arising block matrix systems are addressed [4, 10, 32, 37] and, finally, computational studies are performed.

Numerical analyses of semidiscretizations in time by variational methods and of variational space-time approaches can be found in, for instance [20, 34, 35, 46, 48]. In [48] discontinuous variational approximations of the time variable are studied for abstract parabolic problems whereas in [46] their continuous counterparts are analyzed. In [20, 47] discontinuous variational approximations in time and space are studied and error estimates are proved. In [47] time-dependent domains are considered in an arbitrary Lagrangian Eulerian (ALE) framework and the advection–diffusion equation is written in mixed form as a system of first order equations in space. In [25] a discontinuous Galerkin method in time combined with a stabilized finite element approach in space for first order partial differential equations is investigated for static and dynamically changing meshes. Error estimates in the $L^\infty (L^2)$ and $L^2(L^2)$ norm are derived. In [34, 35] continuous space-time approximations for nonlinear wave equations with mesh modifications and for the Schrödinger equation are considered. Existence and uniqueness of the discrete solutions are discussed and error estimates are proved for the schemes.

As far as the MFE approximation of parabolic problems is concerned, in [48] an error estimate for the semidiscretization in space is given. However, for the flux variable an error estimate is proved only for the $\varvec{L}^2$ norm. No estimate is provided for the error in divergence of the flux, that is part of the natural norm of the underlying function space $\varvec{H}(\mathrm {div};\Omega )$. In [23, 33] similar error estimates, also in negative norms, are presented. In particular, estimates similar to the error estimates for conventional finite element approximations are established. The singular behavior of the error estimates as $t\rightarrow 0$ for initial data in $L^2(\Omega )$ is further included.

In this work a continuous Galerkin–Petrov (cGP) method is used for the discretization in time, whereas the MFEM [17, 21] is applied for the spatial discretization. Appreciable advantages of the MFEM are its local mass conservation property and the inherent approximation of the flux field as part of the formulation itself. In simulating coupled flow and transport processes in porous media the flux approximation of the flow problem is usually of higher practical interest than the approximation of the scalar variable itself. To the best of our knowledge, rigorous error estimates for fully discrete variational space-time discretization schemes that are based on MFE approximations are still missing. In our numerical analysis we split the temporal discretization error from the spatial one by introducing an auxiliary problem based on the semidiscretization in time. We firstly estimate the temporal discretization error and secondly the error between the semidiscrete and the fully discrete solution. The order of convergence estimates are derived in the natural norms of the variational space-time approach. They are summarized in Theorem 4.6. For the scalar variable of the MFE approach one of the given error estimates, measured in the norm of $L^2(0,T;L^2(\Omega ))$, is optimal in space and time if a certain regularity assumption is supposed to be satisfied. For constant scalar-valued diffusion coefficients an error estimate for the flux variable in the norm of $L^2(0,T;\varvec{L}^2(\Omega ))$ is further provided. It is optimal in space and suboptimal in time. In the Gaussian quadrature points of the temporal discretization optimal order error estimates for the flux variable in $\varvec{L}^2(\Omega )$ are even obtained for heterogeneous diffusion matrices. The existence and uniqueness of the semidiscrete and fully discrete solution is further established. Even though a prototype model problem is studied here only, we believe that the techniques for analyzing mixed variational space-time approximation schemes can be applied similarly to more complex flow and transport problems in porous media.

This work is organized as follows. In Sect. 2 our fully discrete variational space-time method is developed. In Sect. 3 we address the semidiscrete problem by proving existence and uniqueness of its solution and error estimates for the semidiscretization in time. In Sect. 4 we study the fully discrete problem and show the existence and uniqueness of its solution. The error between the semidiscrete and fully discrete problem is estimated. In Theorem 4.6 an error estimate for the simultaneous space-time discretization is provided by combining the before-given estimates of the temporal and spatial discretization. In Sect. 5 we illustrate and validate our derived error estimates by numerical experiments. We end our work with some conclusions in Sect. 6.

## The fully discrete variational scheme

### Notation and preliminaries

Throughout this paper, standard notations are used. A summary of the notations used in this work is presented in Appendix B. Let $\Omega \subset \mathbb {R}^d$, with $d=2$ or $d=3$, be a polygonal or polyhedral bounded domain. We denote by $H^p(\Omega )$ the Sobolev space of $L^2$ functions with derivatives up to order *p* in $L^2(\Omega )$ and by $\langle \cdot ,\cdot \rangle$ the inner product in $L^2(\Omega )$. Sobolev spaces of vector-valued functions are written in bold letters. Further, let $H^1_0(\Omega )=\{u\in H^1(\Omega ) \mid u=0 \text{ on } \partial \Omega \}$ and $H^{-1}(\Omega )$ denote its dual space. For the norms of the Sobolev spaces the notation is

$$\begin{aligned} \Vert \cdot \Vert := \Vert \cdot \Vert _{L^2(\Omega )},\quad \Vert \cdot \Vert _p := \Vert \cdot \Vert _{H^p(\Omega )}, \,\, \text{ for } p \in \mathbb {N}, p \ge 1. \end{aligned}$$

For the mixed problem formulation we use the abbreviations

$$\begin{aligned} \varvec{V} = \varvec{H}(\mathrm {div};\Omega )= \left\{ \varvec{q} \in \varvec{L}^2(\Omega ) \mid \nabla \cdot \varvec{q} \in L^2(\Omega )\right\} , \quad W = L^2(\Omega ), \end{aligned}$$

and

$$\begin{aligned} \Vert \varvec{v}\Vert _{\varvec{V}}:= \left( \Vert \varvec{v}\Vert ^2 + \Vert \nabla \cdot \varvec{v} \Vert ^2\right) ^{1/2}. \end{aligned}$$

Let $X_0 \subseteq X \subseteq X_1$ be three reflexive Banach spaces with continuous embeddings. Then we consider the following set of Banach space valued function spaces,

$$\begin{aligned} C(\overline{I};X)&= \left\{ w{:}\,[0,T] \rightarrow X \mid w\,\text{ is } \text{ continuous } \right\} ,\\ L^2(I;X)&= \bigg \{w{:}\,(0,T) \rightarrow X \;\; \bigg |\;\; \int _I \Vert w(t) \Vert _X^2 \; \,\mathrm {d}t < \infty \bigg \},\\ H^1(I;X_0,X_1)&= \left\{ w \in L^2(I;X_0) \mid \partial _t w \in L^2(I;X_1)\right\} , \end{aligned}$$

that are equipped with their naturals norms (cf. [24]) and where the time derivative $\partial _t$ is understood in the sense of distributions on (0, *T*). In particular, every function in $H^1(I;X_0,X_1)$ is continuous on [0, *T*] with values in *X*; cf. [24]. For $X_0=X=X_1$ we simply write $H^1(I;X)$. Moreover, we put $H_0^1(I;X)=\{u\in H^1(I; X)\mid u(0)=0\}$.

For $u\in H^1_0(\Omega )$ let $A{:}\,H^1_0(\Omega )\mapsto H^{-1}(\Omega )$ be defined uniquely by

2.1 $$\begin{aligned} \langle Au, v\rangle = a(u,v) \quad \text{ for } \text{ all }\; v\in H^1_0(\Omega ) \end{aligned}$$

with

$$\begin{aligned} a(u,v):= \langle \varvec{D} \nabla u, \nabla v\rangle , \end{aligned}$$

where the matrix $\varvec{D}=\varvec{D}(\varvec{x})=(d_{ij}(\varvec{x}))_{i,j=1}^d$ satisfies $d_{ij}\in L^\infty (\Omega )$ and is elliptic with

2.2 $$\begin{aligned} D_M | \varvec{\xi }|^2 \ge \varvec{\xi }^\top \varvec{D}(\varvec{x}) \varvec{\xi }\ge D_m |\varvec{\xi }|^2, \quad \theta _M | \varvec{\xi }|^2 \ge \varvec{\xi }^\top \varvec{D}(\varvec{x})^{-1} \varvec{\xi }\ge \theta _m |\varvec{\xi }|^2 , \end{aligned}$$

for almost every $\varvec{x} \in \Omega$, all $\varvec{\xi }\in \mathbb {R}^d$ and some constants $0< D_m \le D_M < \infty$. In (2.2) we put $\theta _m := {D_M}^{-1}$ and $\theta _M := {D_m}^{-1}$. Under the previous assumptions it holds that

2.3 $$\begin{aligned} a(v,v)&\ge \alpha \Vert v\Vert ^2_{1} \quad \text{ for } \text{ all }\; v \in H^1_0(\Omega ), \end{aligned}$$

2.4 $$\begin{aligned} |a(u,v)|&\le \beta \Vert u\Vert _{1}\Vert v\Vert _{1} \quad \text{ for } \text{ all }\; u,v\in H^1_0(\Omega ). \end{aligned}$$

Thus, $A{:}\,H_0^1(\Omega ) \mapsto H^{-1}(\Omega )$ is a linear and continuous operator. For a subspace $D(A) \subset H^1_0(\Omega )$ let $A{:}\,D(A)\mapsto H^{-1}(\Omega )$ be a bijective linear continuous operator. For instance, if $\Omega$ is a convex polygonal or polyhedral bounded domain and $d_{ij}\in W^{1,\infty }(\Omega )$, for $i,j=1,\ldots d$, is satisfied, then the operator *A* is a bijective linear continuous operator from $D(A) = H^2(\Omega )\cap H^1_0(\Omega )$ to $L^2(\Omega )$; cf. [28].

Due to the properties (2.3), (2.4) of the bilinear form $a(\cdot ,\cdot )$ the lemma of Lax–Milgram ensures that the operator $A{:}\,H_0^1(\Omega )\mapsto H^{-1}(\Omega )$ defined in (2.1) is invertible and satisfies in the corresponding operator norm the stability estimates

$$\begin{aligned} \Vert A\Vert _{} \le \beta \quad \text{ and } \quad \Vert A^{-1}\Vert \le \alpha . \end{aligned}$$

Moreover, for all $g\in H^{-1}(\Omega )$ it holds that

2.5 $$\begin{aligned} \langle g, A^{-1} g \rangle = \langle A A^{-1} g, A^{-1}g\rangle \ge \alpha \Vert A^{-1} g\Vert ^2_{1} \ge \frac{\alpha }{\beta ^2}\, \Vert g\Vert ^2_{H^{-1}(\Omega )}. \end{aligned}$$

As usual, by $c>0$ we denote a generic constant throughout the paper.

### Problem formulation

As a prototype model for more sophisticated multiphase flow and multicomponent reactive transport systems in porous media (cf. e.g. [22, 29]) we study in this work

2.6 $$\begin{aligned} \partial _t u - \nabla \cdot (\varvec{D} \nabla u )&= f \quad \mathrm {in}\; \Omega \times I, \end{aligned}$$

2.7 $$\begin{aligned} u&= 0 \quad \mathrm {on}\; \partial \Omega \times I, \end{aligned}$$

2.8 $$\begin{aligned} u(\cdot , 0)&= u_0 \quad \mathrm {in}\; \Omega , \end{aligned}$$

equipped with homogeneous Dirichlet boundary conditions for simplicity only, where $I=(0,T]$ with final time $T>0$ and the diffusion matrix $\varvec{D}$ satisfies the assumptions made in the previous subsection.

Let $f\in L^2(I;W)$ and $u_0\in H^1_0(\Omega )$ be given. Then the existence of a unique weak solution

2.9 $$\begin{aligned} u \in L^2(I;H^1_0(\Omega )) \cap H^1(I;W) \cap C(\overline{I};W) \end{aligned}$$

to (2.6)–(2.8) is ensured; cf. [26, p. 382, Thm. 5]. We note that (2.9) already provides an improved regularity for the weak solution of (2.6)–(2.8); cf. [26, p. 378, Thm. 3].

In order to derive our family of discretization schemes, we first define the auxiliary flux variable $\varvec{q} := - \varvec{D} \nabla u$ for the weak solution *u* of (2.6)–(2.8) that is given by (2.9). Since $\partial _t u \in L^2(I;W)$ is satisfied by (2.9) and $f\in L^2(I;W)$ holds by assumption, it directly follows that $\varvec{q}\in L^2(I;\varvec{V})$. The pair $\{u,\varvec{q}\} \in H^1(I;W)\cap C(\overline{I};W) \times L^2(I;\varvec{V})$ is then also the unique solution to the set of variational equations

2.10 $$\begin{aligned} \int _0^T \langle \partial _t u, w \rangle \,\mathrm {d}t + \int _0^T \langle \nabla \cdot \varvec{q} , w\rangle \,\mathrm {d}t&= \int _0^T \langle f, w \rangle \,\mathrm {d}t , \end{aligned}$$

2.11 $$\begin{aligned} \int _0^T \langle \varvec{D}^{-1} \varvec{q}, \varvec{v}\rangle \,\mathrm {d}t - \int _0^T \langle u, \nabla \cdot \varvec{v} \rangle \,\mathrm {d}t&= 0 \end{aligned}$$

for all $w \in L^2(I;W)$ and $\varvec{v} \in L^2(I;\varvec{V})$ and satisfies the initial condition $u(0)=u_0$. To find (2.11), integration by parts was used. The global problem formulation (2.10), (2.11) motivates our semidiscretization in time.

#### Remark 2.1

- Below, in order apply Lagrange interpolation in time to the function *f*, we need the stronger assumption that $f\in C([0,T];W)$ is satisfied.
- Below, we introduce a semidiscrete approximation in time of the flux $\varvec{q}$ in a subspace of $C([0,T];\varvec{V})$. For this we need to assume that $\varvec{D}\nabla u_0\in \varvec{V}$ holds.
- Higher order regularity of weak solutions to (2.6)–(2.8), that is needed below for the proof of higher order convergence rates, can be obtained under further technical assumptions about the data, coefficients and the boundary of the domain $\Omega$. For the prototype model problem (2.6)–(2.8) such higher order regularity results are well-known; cf. [26, p. 386, Thm. 6]. For (elliptic) regularity results in domains with non-smooth boundaries we refer to, e.g., [28, 39]. Below, we tacitly assume that the required assumptions about the data and $\partial \Omega$ are satisfied such that the existence of a sufficiently regular solution can be assumed. Without such an assumption the application of higher order methods is not meaningful.

### Variational discretization in time by a continuous Galerkin method

For the discretization in time we decompose the time interval (0, *T*] into *N* subintervals $I_n=(t_{n-1},t_n]$, where $n\in \{1,\ldots ,N\}$ and $0=t_0<t_1< \cdots< t_{n-1} < t_n = T$. Further $\tau$ denotes the discretization parameter in time and is defined as the maximum time step size $\tau = \max _{1\le n \le N} \tau _n$, where $\tau _n = t_n-t_{n-1}$. We introduce the function spaces of piecewise polynomials of order *r* in time,

$$\begin{aligned} \mathcal {X}^{r}{(X)}&:=\left\{ u_\tau \in C({\bar{I};\,X}) \;\; \Big | \;\; u_\tau {}_{|{\overline{I}_n}} \in \mathbb {P}_r(\overline{I}_n;\,X),\; \forall n \in \{1,\ldots ,N\} \right\} , \\ \mathcal {Y}^{r}{(X)}&:= \left\{ w_\tau \in L^2({I;\,X}) \;\; \Big | \;\; w_\tau {}_{|{I_n}} \in \mathbb {P}_{r}(I_n;\,X),\; \forall n \in \{1,\ldots ,N\} \right\} , \end{aligned}$$

where

$$\begin{aligned} \mathbb {P}_r(J;\,X) = \bigg \{ p{:}\,J \rightarrow X \;\; \bigg | \;\; p(t) = \sum \limits _{j=0}^{r}{\xi _n^j\, t^j},\; \xi _n^j \in X,\; j=0,\ldots ,r \bigg \} \end{aligned}$$

and $\mathcal {X}^{r}{(X)}\subset H^1(0,T;W)$. We let

$$\begin{aligned} \mathcal {X}_0^{r}{(X)} = \left\{ u_\tau \in \mathcal {X}^{r}{(X)} \;\; \Big | \;\; u_\tau (0) = 0 \right\} . \end{aligned}$$

Further, we put

$$\begin{aligned} \mathcal {W} = X_0^r (W) \times X^r (\varvec{V}) \quad \text{ and } \quad \mathcal {V} = Y^{r-1} (W) \times Y^{r-1} (\varvec{V}). \end{aligned}$$

We equip the function spaces $\mathcal {W}$ and $\mathcal {V}$ with their natural norms being defined by

2.12 $$\begin{aligned} \Vert \{u_\tau ,\varvec{q}_\tau \}\Vert _{\mathcal {W}}^2&= \Vert u_\tau \Vert _{L^2(I;W)}^2 + \Vert \partial _t u_\tau \Vert _{L^2(I;W)}^2 + \Vert \varvec{q}_\tau \Vert ^2_{L^2(I;\varvec{V})}, \nonumber \\ \Vert \{w_\tau ,\varvec{v}_\tau \}\Vert _{\mathcal {V}}^2&= \Vert w_\tau \Vert _{L^2(I;W)}^2 + \Vert \varvec{v}_\tau \Vert ^2_{L^2(I;\varvec{V})}. \end{aligned}$$

With respect to these norms the space $\mathcal {W}$ is a Banach space and the space $\mathcal {V}$ is a reflexive Banach space. Further, we define the space-time bilinear form $a_\tau \in \mathcal {L}(\mathcal {W}\times \mathcal {V};\mathbb {R})$ by means of

$$\begin{aligned} a_\tau \left( \{u_\tau ,\varvec{q}_\tau \},\{w_\tau ,\varvec{v}_\tau \}\right)&= \int _0^T \Big (\langle \partial _t u_\tau , w_\tau \rangle + \langle \nabla \cdot \varvec{q}_\tau , w_\tau \rangle \big ) \,\mathrm {d}t \\&\quad +\,\int _0^T \langle \varvec{D}^{-1} \varvec{q}_\tau , \varvec{v}_\tau \rangle \,\mathrm {d}t - \int _0^T \langle u_\tau , \nabla \cdot \varvec{v}_\tau \rangle \,\mathrm {d}t \end{aligned}$$

for $\{u_\tau ,\varvec{q}_\tau \}\in \mathcal {W}$ and $\{w_\tau ,\varvec{v}_\tau \}\in \mathcal {V}$. Obviously, the mapping $a_\tau {:}\,\mathcal {W} \times \mathcal {V} \mapsto \mathbb {R}$ is linear and continuous, i.e.

2.13 $$\begin{aligned} |a_\tau \left( \{u_\tau ,\varvec{q}_\tau \},\{w_\tau ,\varvec{v}_\tau \}\right) | \le c \Vert \{u_\tau ^0,\varvec{q}_\tau \}\Vert _{\mathcal {W}}\Vert \, \{w_\tau ,\varvec{v}_\tau \}\Vert _{\mathcal {V}} \end{aligned}$$

with some constant $c >0$ independent of $\tau$ and *T*.

For the family of continuous variational time discretization schemes the spaces $\mathcal {X}^{r}{(X)}$ of continuous functions act as spaces for the solution whereas the spaces $\mathcal {Y}^{r-1}{(X)}$ consisting of piecewise polynomials that are discontinuous at the end points of the time intervals are used as test spaces. Since the spaces of the trial and test functions differ here, a discretization of Galerkin–Petrov type is thus obtained.

A semidiscrete variational approximation of the mixed form of problem (2.6)–(2.8), referred as the exact form of cGP(*r*), is then defined by solving the variational equations (2.10), (2.11) in discrete subspaces: *Find* $\{u_\tau , \varvec{q}_\tau \}\in \mathcal {X}^r (W)\times \mathcal {X}^r(\varvec{V})$ *such that*

2.14 $$\begin{aligned}&\int _0^T \langle \partial _t u_\tau ,w_\tau \rangle \,\mathrm {d}t + \int _0^T \langle \nabla \cdot \varvec{q}_\tau , w_\tau \rangle \,\mathrm {d}t = \int _0^T \langle f, w_\tau \rangle \,\mathrm {d}t, \end{aligned}$$

2.15 $$\begin{aligned}&\int _0^T \langle \varvec{D}^{-1} \varvec{q}_\tau , \varvec{v}_\tau \rangle \,\mathrm {d}t - \int _0^T \langle u_\tau , \nabla \cdot \varvec{v}_\tau \rangle \,\mathrm {d}t = 0, \end{aligned}$$

*for all* $w_\tau \in \mathcal {Y}^{r-1}(W)$ *and* $\varvec{v}_\tau \in \mathcal {Y}^{r-1}(\varvec{V})$ *with the initial conditions that* $u_\tau (0) := u_0$ *and* $\varvec{q}_\tau (0) := - \varvec{D}\nabla u_0$ (*cf. Remark* 2.1).

We refer to the solution of Eqs. (2.14), (2.15) as the continuous Galerkin–Petrov method with piecewise polynomials of order *r* and use the notation cGP(*r*). To ensure the existence and uniqueness of solutions to (2.14), (2.15), it is sufficient to use the test spaces $\mathcal {Y}^{r-1}(W)$ and $\mathcal {Y}^{r-1}(\varvec{V})$ with piecewise polynomials of order $r-1$, since the continuity constraint at the discrete time points $t_n$, $n=0,\ldots ,N-1$, that is implied by the definition of the solution spaces $\mathcal {X}^r (W)$ and $\mathcal {X}^r(\varvec{V})$, yields a further condition. By using discontinuous test basis functions $w_\tau (t) = w \psi _{n,i}(t)$ and $\varvec{v}_\tau = \varvec{v} \psi _{n,i}(t)$, for $i=1,\ldots ,r$, with arbitrary time independent functions $w\in W$ and $\varvec{v} \in \varvec{V}$, respectively, and piecewise polynomial functions $\psi _{n,i}{:}\,I\mapsto \mathbb {R}$ that are of order $r-1$ on $I_n$ and vanish on $I\backslash \overline{I}_n$, we can recast the variational equations (2.14), (2.15) as a time marching scheme: *For* $n=1,\ldots , N$ *find* $u_\tau {}_{|\overline{I}_n}\in \mathbb {P}_r(\overline{I}_n;W)$ *and* $\varvec{q}_\tau {}_{|\overline{I}_n}\in P_r(\overline{I}_n;\varvec{V})$ *such that*

2.16 $$\begin{aligned}&\int _{I_n} \langle \partial _t u_\tau , w \rangle \, \psi _{n,i}(t) \,\mathrm {d}t + \int _{I_n} \langle \nabla \cdot \varvec{q}_\tau , w \rangle \, \psi _{n,i}(t) \,\mathrm {d}t = \int _{I_n} \langle f, w\rangle \, \psi _{n,i}(t) \,\mathrm {d}t, \end{aligned}$$

2.17 $$\begin{aligned}&\int _{I_n} \langle \varvec{D}^{-1} \varvec{q}_\tau , \varvec{v} \rangle \, \psi _{n,i}(t) \,\mathrm {d}t - \int _{I_n} \langle u_\tau , \nabla \cdot \varvec{v} \rangle \, \psi _{n,i}(t) \, \,\mathrm {d}t = 0 \end{aligned}$$

*for all* $w\in W$ *and* $\varvec{v} \in \varvec{V}$ *and* $i=1,\ldots ,r$ *with the continuity constraints* $u_\tau {}_{|I_n}(t_{n-1}) = u_\tau {}_{|I_{n-1}}(t_{n-1})$ *and* $\varvec{q}_\tau {}_{|I_n}(t_{n-1}) = \varvec{q}_\tau {}_{|I_{n-1}}(t_{n-1})$ *for* $n\ge 2$ *and the initial conditions* $u_\tau {}_{|I_n}(t_{n-1}) := u_0$, $\varvec{q}_\tau {}_{|I_n}(t_{n-1}) := -\varvec{D}\nabla u_0$ *for* $n = 1$.

To determine $u_\tau {}_{|\overline{I}_n}$ and $\varvec{q}_\tau {}_{|\overline{I}_n}$, we represent them in terms of basis functions, with respect to the time variable, of the spaces $\mathcal {X}^r(W)$ and $\mathcal {X}^r(\varvec{V})$ such that

2.18 $$\begin{aligned} u_\tau {}_{|\overline{I}_n} (t ) = \sum _{j=0}^r U_n^j\, \varphi _{n,j}(t) \quad \mathrm {and} \quad \varvec{q}_\tau {}_{|\overline{I}_n} (t ) = \sum _{j=0}^r \varvec{Q}_n^j \, \varphi _{n,j}(t), \quad \mathrm {for} \; t \in I_n, \end{aligned}$$

with coefficient functions $U_n^j\in W$ and $\varvec{Q}_n^j \in \varvec{V}$ for $j=0,\ldots , r$ and polynomial basis functions $\varphi _{n,j}\in \mathbb {P}_r(\overline{I}_n;\mathbb {R})$ that are Lagrange functions with respect to $r+1$ nodal points $t_{n,j}\in I_n$ satisfying the conditions $\varphi _{n,j} (t_{n,i}) = \delta _{i,j}$ for $i,j=0,\ldots , r$. For the treatment of the continuity constraint in time we put $t_{n,0}=t_{n-1}$. The other points $t_{n,1},\ldots , t_{n,r}$ are chosen as the quadrature points of the *r*-point Gaussian quadrature formula on $I_n$ which is exact if the function to be integrated is a polynomial of degree less or equal to $2r-1$. The basis functions $\varphi _{n,j}\in \mathbb {P}_r(\overline{I}_n;\mathbb {R})$ of (2.18), for $j=0,\ldots , r$, are defined, as usual in the finite element framework, via the affine reference transformation onto $\hat{I} = [0,1]$. The test basis functions $\psi _{n,i}\in P_{r-1}(\overline{I}_n;\mathbb {R})$ with $\psi _{n,i} (t_{n,l}) = \delta _{i,l}$ for $i,l=1,\ldots ,r$ are defined similarly; cf. [15, 37] for details. Now we transform all the time integrals in (2.16), (2.17) to the reference interval $\hat{I}$. By a subsequent application of the *r*-point Gaussian quadrature formula with weights $\hat{\omega }_i$ and quadrature nodes $\hat{t}_i$ on $\hat{I}$ as well as the further notation

$$\begin{aligned} \hat{\alpha }_{ij} := \hat{\omega }_i\cdot \dfrac{\,\mathrm {d}}{\,\mathrm {d}\hat{t}} \hat{\varphi }_j(\hat{t}_i) \quad \text {and} \quad \hat{\beta }_{ij}:= \hat{\omega }_i \cdot \delta _{i,j} \end{aligned}$$

for $i= 1,\ldots ,r$, $j=0,\ldots , r$ (cf. [15, 36, 46]), we obtain the following system of variational problems for the coefficient functions $U_n^j\in W$ and $\varvec{Q}_n^j\in \varvec{V}$ of the representation (2.18): *For* $n=1,\ldots , N$ *and* $j=1,\ldots ,r$ *find coefficient functions* $\{U_n^j,\varvec{Q}_n^j\} \in W\times \varvec{V}$ *such that*

2.19 $$\begin{aligned}&\sum _{j=0}^r \hat{\alpha }_{ij} \langle U_n^j ,w\rangle + {\tau _n} \, \hat{\beta }_{ii} \langle \nabla \cdot \varvec{Q}_n^i ,w\rangle = {\tau _n} \, \hat{\beta }_{ii} \langle f(t_{n,i}) ,w\rangle , \end{aligned}$$

2.20 $$\begin{aligned}&\langle \varvec{D}^{-1} \varvec{Q}_n^i,\varvec{v} \rangle - \langle U_n^i ,\nabla \cdot \varvec{v}\rangle = 0, \end{aligned}$$

*for* $i=1,\ldots , r$ *and all* $\{w,\varvec{v}\}\in W\times \varvec{V}$, *and where due to continuity in time* $U_n^0 = u_\tau {}_{|I_{n-1}}(t_{n-1})$, $\varvec{Q}_n^0 = \varvec{q}_\tau {}_{|I_{n-1}}(t_{n-1})$ *for* $n\ge 2$ *and* $U_n^0:= u_0$, $\varvec{Q}_n^0:=-\varvec{D}\nabla u_0$ *for* $n=1$.

#### Remark 2.2

In the numerical scheme (2.19), (2.20), the flux coefficient functions $\varvec{Q}_n^j$, for $j=1,\ldots , r$, arise only in the *r* Gaussian quadrature points $t_{n,1},\ldots , t_{n,r}\in (t_{n-1},t_n)$ of the subinterval $I_n$. Nevertheless, the coefficient functions $\varvec{Q}_n^0$ for $n\ge 1$, are needed for the unique determination of the semidiscrete flux function $\varvec{q}_\tau \in \mathcal X^r(\varvec{V})$ and an explicit evaluation of $\varvec{q}_\tau {}_{|I_n}$ by the representation (2.18) in other time points of $I_n$ than in the Gaussian quadrature nodes. The fact that the coefficient functions $\varvec{Q}_n^0$ do not arise in (2.19), (2.20) is due to the definition of the Lagrange basis functions $\varphi _{n,j}$ in (2.18) and the fact that the time derivative of the flux variable $\varvec{q}$ does not arise in the model equations.

For the derivation of (2.19), (2.20) from (2.16), (2.17) we tacitly replaced the integrand *f* on the right-hand side by its Lagrange interpolate $\Pi _r f \in \mathbb {P}_r(I_n;L^2(\Omega ))$ defined by

2.21 $$\begin{aligned} \Pi _r f(t)_{|I_n} = \sum _{j=0}^r f(t_{n,j}) \varphi _{n,j}(t) \quad \text{ for }\; t\in I_n. \end{aligned}$$

We note that the constants $\hat{\beta }_{ii}$ are satisfying the following property.

#### Lemma 2.3

[Coefficient property (C)] There exist constants $\beta _m, \beta _M \in \mathbb {R}$ such that

2.22 $$\begin{aligned} 0< \beta _m \le \hat{\beta }_{ii} \le \beta _M < \infty , \quad \text{ for } \; i=1,\ldots ,r, \end{aligned}$$

is satisfied. The constants do not depend on the time step size, but only on the number *r* of involved Gaussian quadrature points.

#### Proof

Indeed, the coefficients $\beta _{ii}= \hat{\omega }_i$ are the Gauss–Legendre quadrature weights

2.23 $$\begin{aligned} \tilde{w}_i = \int _{-1}^1 \mathop {\prod }\limits _{\begin{array}{c} j=1 \\ j \ne i \end{array}}^{r} \left( \frac{x-x_j}{x_i-x_j}\right) ^2 \,\mathrm {d}x= \dfrac{1}{(1 - x_i^2) (P_{r}^\prime (x_i))^2}, \quad i = 1,\ldots ,r, \end{aligned}$$

scaled to the interval [0, 1], i.e. $\hat{\omega }_i = \tilde{\omega }_i/2$. In (2.23), $P_r$ denotes the Legendre polynomial of degree *r* and $x_i$, for $i = 1,\ldots ,r$, are its roots, cf. e.g., [41, p. 436]. Since the sum of the weights $\tilde{\omega }_i$ equals to two and the weights are all strictly positive, we immediately conclude that an upper bound for $\hat{\omega }_i$ is given by one. On the other hand, we know that $| P_r^\prime (x) | \le {r(r+1)}/{2}$ for any $x \in [-1,1]$; cf. [16, p. 73]. This gives us the lower bound $\hat{w}_i \ge 2/(r(r+1))^2$. $\square$

Below, we will also need the following auxiliary results.

#### Lemma 2.4

Let $F(t, \varvec{x}) = \sum _{i=0}^r F_n^i (\varvec{x}) \varphi _{n, i} (t)$, for $t\in I_n$, with coefficient functions $F_n^i \in W$ for $i=0,\ldots ,r$. Then it holds that

2.24 $$\begin{aligned} \sum _{i = 1}^r \sum _{j = 0}^r \hat{\alpha }_{ij} \langle F_{n}^j, F_{n}^i \rangle = \int _{t_{n-1}}^{t_n} \langle \partial _t F, F \rangle dt = \dfrac{1}{2} \Vert F (t_n) \Vert ^2 - \dfrac{1}{2} \Vert F (t_{n-1}) \Vert ^2 \end{aligned}$$

and

2.25 $$\begin{aligned} \Vert F \Vert _{L^2(I_n; W)}^2 \le c\, \tau _n\sum _{j=0}^r \Vert F_{n}^j \Vert _W^2, \end{aligned}$$

for some $c >0$ independent of $\tau _n$. An analogous results holds for coefficients $\varvec{F}_n^i\in \varvec{V}$.

#### Proof

Using the properties of the basis functions $\varphi _i$ and that the *r*-point Gaussian quadrature formula is exact for polynomials of maximum degree $2r - 1$ there holds that

$$\begin{aligned} \int _{t_{n-1}}^{t_n} \langle \partial _t F, F \rangle \,\mathrm {d}t&= \int _{t_{n-1}}^{t_n} \int _\Omega \sum _{j = 0}^r \varphi _{n,j}^\prime (t) F_n^j (\varvec{x}) \sum _{i = 0}^r \varphi _{n,i} (t) F_n^i (\varvec{x}) \,\mathrm {d}\varvec{x} \,\mathrm {d}t \\&= \sum _{j = 0}^r \sum _{i = 0}^r \int _{0}^{1} \frac{\,\mathrm {d}}{\,\mathrm {d}\hat{t}} \hat{\varphi }_{j} (\hat{t}) \cdot \hat{\varphi }_i (\hat{t}) \,\mathrm {d}\hat{t} \; \langle F_n^i, F_n^j \rangle \\&= \sum _{j = 0}^r \sum _{i = 1}^r \hat{w}_i \hat{\varphi }_j^\prime (\hat{t}_{i}) \langle F_n^i, F_n^j \rangle = \sum _{i = 1}^r \sum _{j = 0}^r \hat{\alpha }_{ij} \langle F_{n}^j, F_{n}^i \rangle . \end{aligned}$$

The second of the equalities in (2.24) follows immediately from the first one. It remains to prove (2.25). It holds that

$$\begin{aligned} \Vert F \Vert _{L^2(I_n; W)}^2 \le (r +1) \sum _{i=0}^r \int _{t_{n-1}}^{t_n} \varphi _{n, i}^2 (t) \,\mathrm {d}t \; \Vert F_n^i\Vert ^2 \le c (r +1) \sum _{i=0}^r \tau _n \Vert F_n^i \Vert ^2, \end{aligned}$$

with *c* independent of $\tau _n$. Here we used that $\int _{t_{n-1}}^{t_n} \varphi _{n, i}^2(t) \,\mathrm {d}t \le c\, \tau _n$; cf. [35, p. 1790]. $\square$

### Discretization in space by the mixed finite element method

Now, we present the fully discrete approximation scheme that is obtained by discretizing (2.19), (2.20) with respect to their spatial variables. For this we choose a pair of finite element spaces $W_h \subset W$ and $\varvec{V}_h\subset \varvec{V}$ satisfying the inf–sup stability condition; cf. [17, 21]. Here, we denote by $\mathcal {T}_h=\{K\}$ a finite element decomposition of mesh size *h* of the polyhedral domain $\overline{\Omega }$ into closed subsets *K*, quadrilaterals in two space dimensions and hexahedrals in three space dimensions. Since the software library deal.ii [8] that we use for our implementation of the schemes allows only quadrilateral and hexahedral elements, we restrict ourselves to these types of elements in the following. Triangular and tetrahedral elements can be treated in an analogous way. In our calculations (cf. Sect. 5) we use the Raviart–Thomas element on quadrilateral meshes for two space dimensions. For an application in three dimensions based on the Raviart–Thomas–Nédélec element we refer to [15, 37].

The construction of the discrete function spaces $W_h$ and $\varvec{V}_h$ on quadrilateral and hexahedral finite elements is done by a transformation $\mathcal {T}_K{:}\,\hat{K} \rightarrow K$ of the reference element $\hat{K} = [0,1]^d$, with $d=2$ or $d=3$, to the element *K* through a diffeomorphism $\mathcal {T}_K$ for all $K\in \mathcal {T}_h$. We sketch this briefly for $d=2$; cf. [21, 37] for $d=3$. For this, let

$$\begin{aligned} \hat{Q}^{p_1,p_2} : = \bigg \{\hat{p}: [0,1]^2 \rightarrow \mathbb {R}\;\Big |\; \hat{p} (\varvec{\hat{x}}) = \sum _{i=0}^{p_1} \sum _{j=0}^{p_2} p_{i,j} x_1^i x_2^j, \; p_{i,j}\in \mathbb {R}\bigg \}. \end{aligned}$$

We then define the discrete subspaces $W_h^p \subset W$ and $\varvec{V}_h^p \subset \varvec{V}$ by

2.26 $$\begin{aligned} W_h&= W_h^p := \left\{ w \in W \;\Big | \; w_K \circ \mathcal {T}_K^{-1} \in \hat{Q}^{p,p}, \; \mathrm {for}\; K \in \mathcal {T}_h \right\} , \end{aligned}$$

2.27 $$\begin{aligned} \varvec{V}_h&= \varvec{V}_h^p := \left\{ \varvec{v} \in \varvec{V} \;\Big | \; \varvec{v}_K \circ \mathcal {T}_K^{-1} \in \hat{Q}^{p+1,p}\times \hat{Q}^{p,p+1}, \; \mathrm {for}\; K \in \mathcal {T}_h \right\} . \end{aligned}$$

The fully discrete continuous Galerkin–Petrov and MFE approximation scheme, referred to as cGP(*r*)–MFEM(*p*), then defines fully discrete solutions $u_{\tau ,h}\in \mathcal X^r(W_h)$ and $\varvec{q}_{\tau ,h}\in \mathcal X^r(\varvec{V}_h)$ that are represented in terms of basis functions in time by

$$\begin{aligned} u_{\tau ,h}{}_{|\overline{I}_n} (t ) = \sum _{j=0}^r U_{n,h}^j\, \varphi _{n,j}(t) \quad \mathrm {and} \quad \varvec{q}_{\tau ,h}{}_{|\overline{I}_n} (t ) = \sum _{j=0}^r \varvec{Q}_{n,h}^j \, \varphi _{n,j}(t), \quad \mathrm {for} \; t \in I_n, \end{aligned}$$

with coefficient functions $U_{n,h}^j\in W_h$ and $\varvec{Q}_{n,h}^j \in \varvec{V}_h$ for $j=0,\ldots , r$. The coefficient functions are obtained by solving the variational problem (2.19), (2.20) in the discrete subspaces $W_h\subset W$ and $\varvec{V}_h\subset \varvec{V}$: *For* $n=1,\ldots , N$ *and* $j=1,\ldots , r$ *find coefficient functions* $\{U_{n,h}^j,\varvec{Q}_{n,h}^j\} \in W_h\times \varvec{V}_h$ *such that*

2.28 $$\begin{aligned}&\sum _{j=0}^r \hat{\alpha }_{ij} \langle U_{n,h}^j ,w_h\rangle + {\tau _n} \, \hat{\beta }_{ii} \langle \nabla \cdot \varvec{Q}_{n,h}^i ,w_h\rangle = {\tau _n}\, \hat{\beta }_{ii}\langle f(t_{n,i}) ,w_h\rangle , \end{aligned}$$

2.29 $$\begin{aligned}&\langle \varvec{D}^{-1} \varvec{Q}_{n,h}^i,\varvec{v}_h \rangle - \langle U_{n,h}^i ,\nabla \cdot \varvec{v}_h\rangle = 0 \end{aligned}$$

*for* $i=1,\ldots , r$ *and all* $\{w_h,\varvec{v}_h\}\in W_h\times \varvec{V}_h$, *where* $U_{n,h}^0 \in W_h$ *and* $\varvec{Q}_{n,h}^0 \in \varvec{V}_h$ *are defined by means of the continuity constraint* (*cf. Remark* 2.2), *i.e.*

2.30 $$\begin{aligned} U_{n,h}^0&:= \sum _{j=0}^r U_{n-1,h}^j\, \varphi _{n-1,j}(t_{n-1}) \;\; \mathrm {if}\; n\ge 2 ,&U_{n,h}^0&:= P_h u_0 \;\; \mathrm {if}\; n=1,\nonumber \\ {\varvec{Q}_{n,h}^0}&{ := \sum _{j=0}^r \varvec{Q}_{n-1,h}^j\, \varphi _{n-1,j}(t_{n-1}) \;\; \mathrm {if}\; n\ge 2 ,}&{ \varvec{Q}_{n,h}^0}&{ := \varvec{P}_h (-\varvec{D}\nabla u_0) \;\; \mathrm {if}\; n=1,} \end{aligned}$$

*with* $P_h{:}\,L^2(\Omega )\mapsto W_h$ *and* $\varvec{P}_h{:}\,L^2(\Omega )\mapsto \varvec{V}_h$ *denoting the* $L^2$ *projections onto* $W_h$ *and* $\varvec{V}_h$, *respectively*.

For the derivation of the algebraic formulation of the fully discrete variational problem (2.28), (2.29) we also refer to [15, 36]. In [15, 36], the iterative solution of the arising linear systems and the construction of an efficient preconditioner is further addressed. For solving the algebraic counterpart of Eqs. (2.28), (2.29) we do not apply an additional hybridization technique as it was done, for instance, in [11, 12, 14] and the references therein. We solve the algebraic system by using a Schur complement technique. In [36] the efficiency of the proposed iterative solver along with an adapted preconditioning technique is analyzed numerically. In [15, 36], the approximation properties of some families of space-time discretization schemes, including the cGP(*r*)–MFEM(*p*) approach, in terms of convergence rates and their robustness are studied by numerous numerical experiments. Test cases in three space dimensions and with heterogeneous and strongly anisotropic material properties are also included.

## Existence and uniqueness of the semidiscrete approximation and error estimates

In this subsection we prove the existence and uniqueness of solutions to the semidiscrete approximation scheme that is defined by (2.14), (2.15) and its numerically integrated counterpart (2.19), (2.20), respectively. The time discretization error is also studied in this section. The spatial discretization error is analyzed in Sect. 4.

### Existence and uniqueness of the semidiscrete approximation

#### Theorem 3.1

(Uniqueness of solutions) Let the assumptions of Sect. 2.2 about $\Omega , u_0$ and *f* be satisfied. Then the solution $\{u_\tau ,\varvec{q}_\tau \}\in \mathcal {X}^{r}(W)\times \mathcal {X}^{r}(\varvec{V})$ of the semidiscrete problem (2.14), (2.15) is unique.

#### Proof

Suppose that $\{u_{\tau ,1},\varvec{q}_{\tau ,1}\}\in \mathcal {X}^{r}(W)\times \mathcal {X}^{r}(\varvec{V})$ and $\{u_{\tau ,2},\varvec{q}_{\tau ,2}\}\in \mathcal {X}^{r}(W)\times \mathcal {X}^{r}(\varvec{V})$, respectively, satisfy the semidiscrete problem (2.14), (2.15) and let $u_\tau :=u_{\tau ,1}-u_{\tau ,2}$ and $\varvec{q}_\tau := \varvec{q}_{\tau ,1}-\varvec{q}_{\tau ,2}$. Then, the tuple $\{u_\tau ,\varvec{q}_\tau \}$ satisfies (2.14), (2.15) with $f\equiv 0$. Choosing the test function $w_\tau =A^{-1} \partial _t u_\tau \in \mathcal {Y}^{r-1}(D(A))\subset \mathcal {Y}^{r-1}(W)$ in (2.14) yields that

3.1 $$\begin{aligned} \int _0^T \langle \partial _t u_\tau , A^{-1} \partial _t u_\tau \rangle \,\mathrm {d}t + \int _0^T \langle \nabla \cdot \varvec{q}_\tau , A^{-1} \partial _t u_\tau \rangle \,\mathrm {d}t = 0. \end{aligned}$$

From estimate (2.5) we get that

3.2 $$\begin{aligned} \int _0^T \langle \partial _t u_\tau , A^{-1} \partial _t u_\tau \rangle \,\mathrm {d}t \ge \frac{\alpha }{\beta ^2}\, \int _0^T \Vert \partial _t u_\tau \Vert ^2_{H^{-1}(\Omega )}\,\mathrm {d}t. \end{aligned}$$

Using integration by parts in the second of the integrals in (3.1) and recalling that $A^{-1} \partial _t u_\tau \in D(A)\subset H^1_0(\Omega )$, Eq. (3.1) along with (3.2) yields that

3.3 $$\begin{aligned} 0 \ge \frac{\alpha }{\beta ^2} \int _0^T\Vert \partial _t u_\tau \Vert ^2_{H^{-1}(\Omega )}\,\mathrm {d}t - \int _0^T \langle { \varvec{q}_\tau } , \nabla A^{-1} \partial _t u_\tau \rangle \,\mathrm {d}t. \end{aligned}$$

Next, by choosing the test function $\varvec{v}_\tau = \varvec{D} \nabla A^{-1} \partial _t u_\tau \in \mathcal {Y}^{r-1}(\varvec{V})$ in Eq. (2.15) we find that

3.4 $$\begin{aligned} \int _0^T \langle \varvec{D}^{-1} \varvec{q}_\tau , \varvec{D} \nabla A^{-1} \partial _t u_\tau \rangle \,\mathrm {d}t - \int _0^T \langle u_\tau , \nabla \cdot (\varvec{D} \nabla A^{-1} \partial _t u_\tau ) \rangle \,\mathrm {d}t = 0. \end{aligned}$$

Since

$$\begin{aligned} \nabla \cdot (\varvec{D} \nabla A^{-1} \partial _t u_\tau ) = - A A^{-1}\partial _t u_\tau = - \partial _t u_\tau \end{aligned}$$

and $\varvec{D} = \varvec{D}^\top$ by assumption, it follows from (3.4) that

$$\begin{aligned} \int _0^T \langle \varvec{q}_\tau , \nabla A^{-1} \partial _t u_\tau \rangle \,\mathrm {d}t + \dfrac{1}{2}\int _0^T \dfrac{\,\mathrm {d}}{\,\mathrm {d}t} \Vert u_\tau \Vert ^2 \,\mathrm {d}t = 0. \end{aligned}$$

Since $u_\tau (0) = u_{\tau ,1} (0) - u_{\tau ,2} (0) = 0$ it follows that

3.5 $$\begin{aligned} \int _0^T \langle \varvec{q}_\tau , \nabla A^{-1} \partial _t u_\tau \rangle \,\mathrm {d}t + \dfrac{1}{2} \Vert u_\tau (T) \Vert ^2 = 0. \end{aligned}$$

Combing relations (3.3) and (3.5) shows that

3.6 $$\begin{aligned} 0 \ge c \int _0^T\Vert \partial _t u_\tau \Vert ^2_{H^{-1}(\Omega )}\,\mathrm {d}t + \dfrac{1}{2} \Vert u_\tau (T) \Vert ^2 \end{aligned}$$

and, therefore, $u_\tau =0$. This implies the uniqueness of solutions $u_\tau$ to (2.14), (2.15).

To show the uniqueness of solutions $\varvec{q}_\tau$ of (2.14), (2.15), we choose the test function $\varvec{v}_\tau = \partial _t \varvec{q}_\tau \in \mathcal {Y}^{r-1}(\varvec{V})$. Recalling that $u_\tau =0$ by means of the uniqueness result (3.6) we obtain from Eq. (2.15) that

$$\begin{aligned} \int _0^T \langle \varvec{D}^{-1} \varvec{q}_\tau , \partial _t \varvec{q}_\tau \rangle \,\mathrm {d}t = 0. \end{aligned}$$

From $\langle \varvec{D}^{-1}\varvec{q}_\tau , \partial _t \varvec{q}_\tau \rangle = \frac{1}{2} \frac{\,\mathrm {d}}{\,\mathrm {d}t}\langle \varvec{D}^{-1} \varvec{q}_\tau , \varvec{q}_\tau \rangle$ and $\varvec{q}_\tau (0) = \varvec{0}$ we conclude that

3.7 $$\begin{aligned} 0 =\frac{1}{2} \Vert \varvec{D}^{-1/2} \varvec{q}_\tau (T)\Vert ^2. \end{aligned}$$

Next, we choose $\varvec{v}_\tau = \partial _t^2 \varvec{q}_\tau \in \mathcal {Y}^{r-2}(\varvec{V})$, $\mathcal {Y}^{r-2}(\varvec{V})\subset \mathcal {Y}^{r-1}(\varvec{V})$ by definition, in (2.15), recall that $u_\tau =0$ and use that

$$\begin{aligned} \dfrac{\,\mathrm {d}}{\,\mathrm {d}t} \langle \varvec{D}^{-1} \varvec{q}_\tau , \partial _t \varvec{q}_\tau \rangle = \langle \varvec{D}^{-1} \partial _t \varvec{q}_\tau , \partial _t \varvec{q}_\tau \rangle + \langle \varvec{D}^{-1} \varvec{q}_\tau , \partial _t^2 \varvec{q}_\tau \rangle . \end{aligned}$$

Together, this implies that

3.8 $$\begin{aligned} 0 = \int _0^T \dfrac{\,\mathrm {d}}{\,\mathrm {d}t} \langle \varvec{D}^{-1} \varvec{q}_\tau , \partial _t \varvec{q}_\tau \rangle \,\mathrm {d}t - \int _0^T \Vert \varvec{D}^{-1/2} \partial _t \varvec{q}_\tau \Vert ^2 \,\mathrm {d}t. \end{aligned}$$

Since $\varvec{q}_\tau (0)=\varvec{q}_{\tau ,1}(0)-\varvec{q}_{\tau ,2}(0) = \varvec{0}$ and, further, $\varvec{q}_\tau (T)= \varvec{0}$ by means of (3.7), it follows from Eq. (3.8) along with property (3.7) that $\varvec{q}_\tau = 0$. The uniqueness of solutions to the variational problem (2.14), (2.15) is thus proved. $\square$

#### Theorem 3.2

(Existence of solutions) Let the assumptions of Sect. 2.2 about $\Omega , u_0, \varvec{D}$ and *f* be satisfied. Then the semidiscrete problem (2.14), (2.15) admits a solution $\{u_\tau ,\varvec{q}_\tau \}\in \mathcal {X}^{r}(W)\times \mathcal {X}^{r}(\varvec{V})$.

#### Proof

To prove existence of solutions to problem (2.14), (2.15), we will use an equivalent conformal formulation, see [43] for a similar approach.

*Find* $\widetilde{u}_\tau \in X^r (H^1_0(\Omega ))$ *such that* $\widetilde{u}_\tau (0) = u_0$ *and*

3.9 $$\begin{aligned} \int _0^T \langle \partial _t \widetilde{u}_\tau , w_\tau \rangle \,\mathrm {d}t + \int _0^T a(\widetilde{u}_\tau ,w_\tau ) \,\mathrm {d}t = \int _0^T \langle f,w_\tau \rangle \,\mathrm {d}t \end{aligned}$$

*for all* $$w_\tau \in Y^{r-1}(H^1_0(\Omega ))$$.

The existence and uniqueness of the semidiscrete approximation satisfying (3.9) can be established. This is shown in the “Appendix” of this work. Then we define

3.10 $$\begin{aligned} u_\tau := \widetilde{u}_\tau \quad \text{ and } \quad \varvec{q}_\tau := - \varvec{D} \nabla \widetilde{u}_\tau . \end{aligned}$$

Obviously, it holds that $u_\tau \in X^r(W)$ since $H^1_0(\Omega )\subset W$. Further, we have that $\partial _t \widetilde{u}_\tau \in L^2(I;H^1_0(\Omega ))$ since on each of the subintervals $I_n$, $n=1,\ldots , N$ the function $\widetilde{u}_\tau \in X^r(H^1_0(\Omega ))$ admits the representation

$$\begin{aligned} u_\tau {}_{|I_n} (t) = \sum _{j=0}^r U_n^j \varphi _{n,j}(t), \quad \text{ for }\; t\in I_n, \end{aligned}$$

with coefficients $U_n^j \in H^1_0(\Omega )$ and polynomial basis functions $\varphi _{n,j}\in \mathbb {P}_r(I_n;\mathbb {R})$.

Next, we prove that $\varvec{q}_\tau \in X^r(\varvec{V})$. Under the assumption of Sect. 2.2 that $f\in L^2(I;W)$ it follows that

$$\begin{aligned} \int _0^T \langle - \varvec{q}_\tau , \nabla w_\tau \rangle \,\mathrm {d}t&= \int _0^T \langle \varvec{D} \nabla u_\tau , \nabla w_\tau \rangle \,\mathrm {d}t\\&= \int _0^T \langle f-\partial _t u_\tau ,w_\tau \rangle \,\mathrm {d}t =:\int _0^T \langle \widetilde{f} ,w_\tau \rangle \,\mathrm {d}t \end{aligned}$$

for all $w_\tau \in Y^{r-1} (C_0^\infty (\Omega ))$ with $\widetilde{f} \in L^2(I;L^2(\Omega ))$. Thus, we have that

$$\begin{aligned} \int _0^T \langle - \varvec{q}_\tau , \nabla w_\tau \rangle \,\mathrm {d}t =\int _0^T \langle \widetilde{f} ,w_\tau \rangle \,\mathrm {d}t. \end{aligned}$$

Consequently, it holds that (cf. [17, p. 18, Eq. (3.38)])

$$\begin{aligned} \int _0^T \langle \nabla \cdot \varvec{q}_\tau , w_\tau \rangle \,\mathrm {d}t =\int _0^T \langle \widetilde{f} ,w_\tau \rangle \,\mathrm {d}t \end{aligned}$$

for all $w_\tau \in Y^{r-1} (C_0^\infty (\Omega ))$ in the sense of distributions. Since $\widetilde{f} \in L^2(I;L^2(\Omega ))$, it follows that $\nabla \cdot \varvec{q}_\tau \in L^2(I;L^2(\Omega ))$ and, therefore, that $\varvec{q}_\tau \in L^2(I; \varvec{V})$ is fulfilled. Finally, from the expansion in terms of polynomial basis functions

3.11 $$\begin{aligned} \varvec{q}_\tau (t) = - \sum _{j=0}^r \varvec{D} \nabla U_n^j \, \varphi _{n,j}(t), \end{aligned}$$

we conclude that $\varvec{q}_\tau \in C([0,T];\varvec{V})$.

Equation (3.9) then directly implies that the functions $u_\tau$ and $\varvec{q}_\tau$ defined in (3.10) satisfy the first equation of the variational problem (2.14), (2.15). The second equation of the system (2.14), (2.15) then follows from the representation (3.11) of the variable $\varvec{q}_\tau$ by testing the identity (3.11) with some function $\varvec{v}_\tau \in \mathcal {Y}^{r-1}(\varvec{V})$ and applying the divergence theorem of Gauss. Hence, the assertion of the theorem is proved. $\square$

As a corollary of the previous two theorems proving the existence of a unique solution to the semidiscrete problem (2.14), (2.15) we obtain an inf–sup stability condition within our space-time framework. This result will play a fundamental role in our error analyses. For this we need some further notation. Let $\{u_\tau ,\varvec{q}_\tau \}\in \mathcal {X}^{r}(W)\times \mathcal {X}^{r}(\varvec{V})$ denote the solution of the semidiscrete problem (2.14), (2.15). We split $u_\tau$ as

3.12 $$\begin{aligned} u_\tau (t) = u_0 + u^0_\tau (t) \quad \text {with}\quad u^0_\tau \in \mathcal {X}_0^{r}(W). \end{aligned}$$

In terms of the tuple $\{u^0_\tau , \varvec{q}_\tau \}$ of unknowns we recast the existence and uniqueness result of Theorems 3.1 and 3.2 in the following form.

#### Corollary 3.3

Let the assumptions of Sect. 2.2 about $\Omega , u_0, \varvec{D}$ and *f* be satisfied. Let $\{u_\tau ,\varvec{q}_\tau \}\in \mathcal {X}^{r}(W)\times \mathcal {X}^{r}(\varvec{V})$ be the unique solution of the semidiscrete problem (2.14), (2.15) according to Theorems 3.1 and 3.2. Then, the tuple $\{u_\tau ^0,\varvec{q}_\tau \}\in \mathcal {X}_0^{r}(W)\times \mathcal {X}^{r}(\varvec{V})$ with $u_\tau ^0$ being defined in (3.12) is the unique solution of the following variational problem: *Find* $\{u^0_\tau , \varvec{q}_\tau \}\in \mathcal {X}_0^r (W)\times \mathcal {X}^r(\varvec{V})$ *such that*

3.13 $$\begin{aligned} \int _0^T \langle \partial _t u^0_\tau ,w_\tau \rangle \,\mathrm {d}t + \int _0^T \langle \nabla \cdot \varvec{q}_\tau , w_\tau \rangle \,\mathrm {d}t&= \int _0^T \langle f, w_\tau \rangle \,\mathrm {d}t, \end{aligned}$$

3.14 $$\begin{aligned} \int _0^T \langle \varvec{D}^{-1} \varvec{q}_\tau , \varvec{v}_\tau \rangle \,\mathrm {d}t - \int _0^T \langle u^0_\tau , \nabla \cdot \varvec{v}_\tau \rangle \,\mathrm {d}t&= \int _0^T \langle u_0 , \nabla \cdot \varvec{v}_\tau \rangle \,\mathrm {d}t \end{aligned}$$

*for all* $w_\tau \in \mathcal {Y}^{r-1}(W)$ *and* $\varvec{v} \in \mathcal {Y}^{r-1}(\varvec{V})$.

As a corollary we get the following inf–sup stability condition.

#### Corollary 3.4

Let the assumptions of Sect. 2.2 about $\Omega , u_0, \varvec{D}$ and *f* be satisfied. Then, there exists a constant $\gamma > 0$ such that

3.15 $$\begin{aligned} \inf _{\{u^0_\tau ,\varvec{q}_\tau \}\in \mathcal {W}\backslash \{\varvec{0}\}} \sup _{\{w_\tau ,\varvec{v}_\tau \}\in \mathcal {V}\backslash \{\varvec{0}\}} \frac{a_\tau \left( \{u^0_\tau ,\varvec{q}_\tau \},\{w_\tau ,\varvec{v}_\tau \}\right) }{\Vert \{u^0_\tau ,\varvec{q}_\tau \}\Vert _{\mathcal {W}} \, \Vert \{w_\tau ,\varvec{v}_\tau \}\Vert _{\mathcal {V}}} \ge \gamma > 0. \end{aligned}$$

#### Proof

The discrete problem (3.13), (3.14) satisfies the assumptions of the Banach–Nečas–Babuška theorem [24, p. 85]. Since the discrete problem (3.13), (3.14) is well-posed according to Corollary 3.3, the Banach–Nečas–Babuška theorem implies the inf–sup stability condition (3.15). $\square$

### Estimates for the error between the continuous and the semidiscrete solution

Now we shall show error estimates for the exact form (2.14), (2.15) of the cGP(*r*) approach applied to the mixed formulation (2.10), (2.11) of our parabolic model problem.

For this we assume that the following approximation property are satisfied. There exist interpolation operators $I_\tau {:}\,H^1_0(I,W) \mapsto \mathcal {X}_0^r(W)$, $\varvec{J}_\tau {:}\,L^2(I;\varvec{V})\mapsto \mathcal {X}^r(\varvec{V})$ such that for sufficiently smooth functions $u\in H^1(I;W)$ and $\varvec{q} \in L(I;\varvec{V})$ and all time intervals $I_n$, for $n=1,\ldots ,N$, it holds that

3.16 $$\begin{aligned} \Vert u - I_\tau u\Vert _{L^2(I_n;W)}&\le c\, \tau _n^{r+1} \Vert \partial _t^{r+1} u\Vert _{L^2(I_n;W)}, \end{aligned}$$

3.17 $$\begin{aligned} \Vert \partial _t(u - I_\tau u)\Vert _{L^2(I_n;W)}&\le c\, \tau _n^{r} \Vert \partial _t^{r+1} u\Vert _{L^2(I_n;W)}, \end{aligned}$$

3.18 $$\begin{aligned} \Vert \varvec{q} - \varvec{J}_\tau \varvec{q}\Vert _{L^2(I_n;\varvec{V})}&\le c\, \tau _n^{r+1} \Vert \partial _t^{r+1} \varvec{q}\Vert _{L^2(I_n;\varvec{V})} \end{aligned}$$

with some constant *c* independent of $\tau _n$ and $\tau$. The existence of such approximations is obviously ensured, for instance, by using Lagrange interpolation [48].

We get the following error estimates in the natural norm of the time discretization.

#### Theorem 3.5

[Space-time error estimate for exact form of cGP(*r*)] Let the assumptions of Sect. 2.2 about $\Omega , u_0, \varvec{D}$ and *f* be satisfied. Let $\{u,\varvec{q}\} \in H^1(I;W)\times L^2(I;\varvec{V})$ denote the unique solution of the mixed problem (2.10), (2.11) that is supposed to be sufficiently regular. Then the solution $\{u_\tau ,\varvec{q}_\tau \}\in \mathcal {X}^{r}(W)\times \mathcal {X}^{r}(\varvec{V})$ of the semidiscrete problem (2.14), (2.15) satisfies the error estimate

$$\begin{aligned} \Vert \{u - u_\tau ,\varvec{q} - \varvec{q}_\tau \}\Vert _{\mathcal {W}}&\le c \left\{ \sum _{n=1}^N \tau _n^{2r} \Big (\Vert \partial _t^{r+1} u\Vert ^2_{L^2(I_n;W)} + \Vert \partial _t^{r+1} \varvec{q} \Vert ^2_{L^2(I_n;\varvec{V})}\Big ) \right\} ^{1/2}\\&\le c \tau ^{r} \Big (\Vert \partial _t^{r+1} u\Vert _{L^2(I;W)}+\Vert \partial _t^{r+1} \varvec{q} \Vert _{L^2(I;\varvec{V})}\Big ) , \end{aligned}$$

where the constant *c* is independent of $\tau _n$, $\tau$ and *T*.

#### Proof

By splitting

3.19 $$\begin{aligned} u(t) = u_0 + u^0(t) \quad \mathrm {with}\quad u^0\in H^1_0(I;W) \end{aligned}$$

and recalling the semidiscrete counterpart (3.12), we get that

$$\begin{aligned} u(t) - u_\tau (t) = u^0(t) - u_\tau ^0(t), \quad \partial ^r_t u(t) = \partial _t^r u^0(t) \end{aligned}$$

for almost every $t\in (0,T)$, such that it is sufficient to derive the asserted error bounds of the theorem for $u^0-u_\tau ^0$ instead of estimating $u-u_\tau$. This will be done in the following.

By (3.16)–(3.18) it holds that

3.20 $$\begin{aligned}&\Vert \{u^0- I_\tau u^0,\varvec{q}- \varvec{J}_\tau \varvec{q}\}\Vert _{\mathcal {W}} \nonumber \\&\quad \le c \left\{ \sum _{n=1}^N \tau _n^{2r} \Big (\Vert \partial _t^{r+1} u^0\Vert ^2_{L^2(I_n;W)} + \Vert \partial _t^{r+1} \varvec{q} \Vert ^2_{L^2(I_n;\varvec{V})}\Big ) \right\} ^{1/2}\nonumber \\&\quad \le c \tau ^{r} \Big (\Vert \partial _t^{r+1} u^0\Vert _{L^2(I;W)}+\Vert \partial _t^{r+1} \varvec{q} \Vert _{L^2(I;\varvec{V})}\Big ). \end{aligned}$$

For the discrete functions $w_\tau := u^0_\tau - I_\tau u^0\in \mathcal {X}_0^r(W)$, $\varvec{v}_\tau = \varvec{q}_\tau - \varvec{J}_\tau \varvec{q}\in \mathcal {X}^r(\varvec{V})$ there exist, due to the inf–sup stability condition (3.15), functions $\varphi _\tau \in \mathcal {X}_0^r(W)$, $\varvec{\psi }_\tau \in \mathcal {X}^r(\varvec{V})$ such that

3.21 $$\begin{aligned} \gamma \Vert \{w_\tau ,\varvec{v}_\tau \} \Vert _{\mathcal {W}} \Vert \{\varphi _\tau { ,}\varvec{\psi }_\tau \} \Vert _{\mathcal {V}}&\le a_\tau \left( \{w_\tau ,\varvec{v}_\tau \},\{\varphi _\tau ,\varvec{\psi }_\tau \}\right) \nonumber \\&= a_\tau \left( \{u^0 - I_\tau u^0,\varvec{q} - \varvec{J}_\tau \varvec{q}\},\{\varphi _\tau ,\varvec{\psi }_\tau \}\right) \nonumber \\&\le c \Vert \{u^0 - I_\tau u^0,\varvec{q} - \varvec{J}_\tau \varvec{q}\} \Vert _{\mathcal {W}} \Vert \{\varphi _\tau ,\varvec{\psi }_\tau \} \Vert _{\mathcal {V}} , \end{aligned}$$

where the Galerkin orthogonalities

$$\begin{aligned} \int _0^T \langle \partial _t (u^0_\tau -u^0) ,w_\tau \rangle \,\mathrm {d}t + \int _0^T \langle \nabla \cdot (\varvec{q}_\tau - \varvec{q}), w_\tau \rangle \,\mathrm {d}t&= 0,\\ \int _0^T \langle \varvec{D}^{-1} (\varvec{q}_\tau - \varvec{q}) , \varvec{v}_\tau \rangle \,\mathrm {d}t - \int _0^T \langle u^0_\tau - u^0 , \nabla \cdot \varvec{v}_\tau \rangle \,\mathrm {d}t&= 0 \end{aligned}$$

have been used. From (3.21) along with (3.20), we find that

3.22 $$\begin{aligned}&\Vert \{u^0_\tau - I_\tau u^0,\varvec{q}_\tau - \varvec{J}_\tau \varvec{q}\}\Vert _{\mathcal {W}} \le {c}{\gamma }^{-1} \Vert \{u^0- I_\tau u^0,\varvec{q}- \varvec{J}_\tau \varvec{q}\}\Vert _{\mathcal {W}}\nonumber \\&\quad \le {c }{\gamma }^{-1} \, \tau ^{r} \, \Big (\Vert \partial _t^{r+1} u\Vert _{L^2(I;W)}+\Vert \partial _t^{r+1} \varvec{q} \Vert _{L^2(I;\varvec{V})}\Big ) . \end{aligned}$$

From inequality (3.22) along with the interpolation error estimate (3.20) we conclude the assertion of the theorem by means of the triangle inequality. $\square$

Theorem 3.5 yields an error estimate with respect to the natural space-time norm of the discretization scheme. The estimate is sharp with respect to the contribution of $\Vert \partial _t (u- u_\tau )\Vert _{L^2(I;W)}$ to the overall norm (2.12). However, the estimate is suboptimal with respect to $\Vert u- u_\tau \Vert _{L^2(I;W)}$. In the following theorem, we sharpen our analysis by providing an optimal order error estimate also for $\Vert u- u_\tau \Vert _{L^2(I;W)}$. This is done by a duality argument. For this, the following additional regularity assumption is needed.

**Regularity condition** (${\mathbf {\mathbf{R}}}_{\mathbf {\mathbf{mix}}}$) *Suppose that* $g\in L^2(I;W)$. *The variational problem, find* $z\in H^1(I;W)$ and $\varvec{p} \in L^2(I;\varvec{V})$ *with* $z(T)=0$ *such that*

3.23 $$\begin{aligned} \int _0^T \big (\langle - \partial _t z , w \rangle + \langle \nabla \cdot \varvec{p} , w \rangle \big ) \,\mathrm {d}t&= \int _0^T \langle g,w\rangle \,\mathrm {d}t, \end{aligned}$$

3.24 $$\begin{aligned} \int _0^T \big ( \langle \varvec{D}^{-1} \varvec{p},\varvec{v} \rangle - \langle z, \nabla \cdot \varvec{v}\rangle \big ) \,\mathrm {d}t&= 0 \end{aligned}$$

*for all* $w\in L^2(T,0;W)$, $\varvec{v} \in L^2(T,0;\varvec{V})$ *admits a unique solution* $\{z,\varvec{p}\} \in H^1(I;L^2(\Omega ))$ $\times L^2(I;\varvec{V})$ *with the improved regularity* $\varvec{p} \in H^1(I;\varvec{V})$ *and the a priori estimate*

3.25 $$\begin{aligned} \Vert \partial _t \varvec{p} \Vert _{L^2(I;\varvec{V})} \le c \Vert g\Vert _{L^2(I;W)}. \end{aligned}$$

Formally, the corresponding strong form of (3.23), (3.24) is given by

3.26 $$\begin{aligned} - \partial _t z + \nabla \cdot \varvec{p} = g , \quad \varvec{D}^{-1} \varvec{p} + \nabla z = 0 \quad \text{ in } \Omega \times (0,T) , \end{aligned}$$

with $z(T)=0$ and homogeneous Dirichlet boundary conditions, that is obtained by rewriting the dual problem associated with (2.6)–(2.8),

3.27 $$\begin{aligned} -\partial _t z - \nabla \cdot (\varvec{D}\nabla z) = g\; \text{ in } \Omega \times (0,T) , \quad z(T) = 0\; \text{ in } \Omega , \quad z = 0 \; \text{ on } \partial \Omega \times (0,T), \end{aligned}$$

as a system of first order equations. Defining the transformation $\widetilde{z}(t):=z(T-t)$ and $\widetilde{g}(t):=z(T-t)$ we recast (3.27) as a forward parabolic problem in $\widetilde{z}$,

$$\begin{aligned} \partial _t \widetilde{z} - \nabla \cdot (\varvec{D}\nabla \widetilde{z}) = \widetilde{g}\; \text{ in } \Omega \times I , \quad \widetilde{z}(0) = 0\; \text{ in } \Omega , \quad \widetilde{z} = 0 \; \text{ on } \partial \Omega \times (0,T), \end{aligned}$$

such that standard existence and stability estimates can be applied; cf. [26, p. 382, Theorem 5]. Then, defining the variable $\varvec{p}$ by means of the second of the identities in (3.26), the thus obtained tuple $\{z,\varvec{p}\}$ satisfies the variational problem (3.23), (3.24). Moreover, for $g \in L^2(I;W)$, from [26, p. 382, Theorem 5] we get the a priori estimate

3.28 $$\begin{aligned} \Vert \partial _t z \Vert _{L^2(I;W)} + \Vert \varvec{p} \Vert _{L^2(I;\varvec{V})} \le c \Vert g \Vert _{L^2(I;W)}. \end{aligned}$$

For this we note that $\varvec{p} \in L^2(I;\varvec{V})$ can be shown by using the arguments of the proof of Theorem 3.2. The a priori estimate of the vector variable $\varvec{p}$ in (3.28) is then a direct consequence of the variational equation (3.23).

#### Remark 3.6

A regularity condition similar to (R${}_{\mathrm {mix}}$) is also used in [46, p. 48, Eq. (6.16)] to prove the optimal order convergence of a variational time discretization of second order parabolic problems in the non-mixed formulation. Currently, it remains an open problem how this limiting condition can be avoided in the theoretical analysis. The techniques that were developed recently in [25] might be helpful. However, in our numerical convergence studies of Sect. 5 the optimal convergence rate that is proved in Theorem 3.8 under the condition (R${}_{\mathrm {mix}}$) is nicely observed.

Below we also need the following auxiliary lemma.

#### Lemma 3.7

Let $I_0{:}\,H^1(I,W)\mapsto \mathcal {Y}^0(W)$ and $\varvec{J}_0{:}\,H^1(I,\varvec{V})\mapsto \mathcal {Y}^0(\varvec{V})$ be interpolation operators that are defined on each subinterval $I_n$ by means of

$$\begin{aligned} I_0 u(t) := u(t_{n-1}) \quad and \quad \varvec{J}_0 \varvec{v}(t) := \varvec{v}(t_{n-1}) \quad \text {for all}\;\; t \in I_n. \end{aligned}$$

Then it holds that

3.29 $$\begin{aligned} \Vert z-I_0 z\Vert _{L^2(I_n,W)}&\le \tau _n \Vert \partial _t z \Vert _{L^2(I_n;W)}, \end{aligned}$$

3.30 $$\begin{aligned} \Vert \varvec{p} -\varvec{J}_0 \varvec{p} \Vert _{L^2(I_n;\varvec{V})}&\le \tau _n \Vert \partial _t \varvec{p} \Vert _{L^2(I_n;\varvec{V})}\, . \end{aligned}$$

#### Proof

The assertions directly follow from [46, Lemma 6.2]; cf. also [46, p. 49]. $\square$

#### Theorem 3.8

$[L^2$ Error estimate for the exact form of cGP(*r*)] Let the assumptions of Sect. 2.2 about $\Omega , u_0, \varvec{D}$ and *f* be satisfied. Further, suppose that the regularity condition (R${}_{\mathrm {mix}})$ holds. Let $\{u,\varvec{q}\} \in H^1(I;W)\times L^2(I;\varvec{V})$ denote the unique solution of the mixed problem (2.10), (2.11) that is supposed to be sufficiently regular. Then the solution $\{u_\tau ,\varvec{q}_\tau \}\in \mathcal {X}^{r}(W)\times \mathcal {X}^{r}(\varvec{V})$ of the semidiscrete problem (2.14), (2.15) satisfies the error estimate

$$\begin{aligned} \Vert u - u_\tau \Vert _{L^2(I;W)}&\le c \, \tau \left\{ \sum _{n=1}^N \tau _n^{2r} \Big (\Vert \partial _t^{r+1} u\Vert ^2_{L^2(I_n;W)} + \Vert \partial _t^{r+1} \varvec{q} \Vert ^2_{L^2(I_n;\varvec{V})}\Big ) \right\} ^{1/2}\\&\le c \, \tau ^{r+1} \Big (\Vert \partial _t^{r+1} u\Vert _{L^2(I;W)}+\Vert \partial _t^{r+1} \varvec{q} \Vert _{L^2(I;\varvec{V})}\Big ). \end{aligned}$$

#### Proof

We put $e_u:=u^0 -u^0_\tau \in L^2(I;W)$ and $\varvec{e}_{\varvec{q}}:= \varvec{q} - \varvec{q}_\tau \in L^2(I;\varvec{V})$ with the splitting (3.19) and (3.12) of the scalar variable and its semidiscrete approximation, respectively. Further, let $\{z,\varvec{p}\}\in H^1(0,T;W)\cap C([0,T];W) \times L^2(0,T;\varvec{V})$ with $z(T)=0$ denote the unique solution of (3.23), (3.24) with right-hand side function $g=e_u$.

Firstly, recalling that $z(T)=0$ and $e_u(0)=0$ by definition, we get that

3.31 $$\begin{aligned} \int _0^T \langle - \partial _t z , e_u \rangle \,\mathrm {d}t = -{z(T)}e_u(T)+ z(0){e_u(0)} + \int _0^T \langle \partial _t e_u ,z\rangle \,\mathrm {d}t= \int _0^T \langle \partial _t e_u ,z\rangle \,\mathrm {d}t. \end{aligned}$$

Choosing the test function $w=e_u$ in (3.23) and using (3.31), we find that

3.32 $$\begin{aligned}&\int _0^T \Vert e_u \Vert ^2 \,\mathrm {d}t = \int _0^T \big (\langle \partial _t e_u , z\rangle + \langle \nabla \cdot \varvec{p}, e_u \rangle \big )\,\mathrm {d}t \nonumber \\&\quad = \int _0^T \big (\langle \partial _t e_u , z\rangle + \langle \nabla \cdot \varvec{e}_{\varvec{q}}, z \rangle \big )\,\mathrm {d}t -\int _0^T \big (\langle \nabla \cdot \varvec{e}_q, z \rangle - \langle \nabla \cdot \varvec{p}, e_u \rangle \big )\,\mathrm {d}t. \end{aligned}$$

Choosing the test function $\varvec{v}=\varvec{e}_{\varvec{q}}$ in (3.24) and recalling that the matrix $\varvec{D}$ is symmetric by assumption, we conclude that

3.33 $$\begin{aligned} \int _0^T \langle \nabla \cdot \varvec{e}_{\varvec{q}}, z \rangle \,\mathrm {d}t = \int _0^T \langle \varvec{D}^{-1} \varvec{p}, \varvec{e}_{\varvec{q}}\rangle \,\mathrm {d}t = \int _0^T \langle \varvec{D}^{-1} \varvec{e}_{\varvec{q}}, \varvec{p} \rangle \,\mathrm {d}t. \end{aligned}$$

From (3.32) and (3.33) it then follows that

3.34 $$\begin{aligned} \int _0^T \Vert e_u \Vert ^2 \,\mathrm {d}t =\int _0^T \big (\langle \partial _t e_u , z\rangle + \langle \nabla \cdot \varvec{e}_q, z \rangle \big )\,\mathrm {d}t - \int _0^T \big (\langle \varvec{D}^{-1} \varvec{e}_{\varvec{q}}, \varvec{p} \rangle - \langle e_u, \nabla \cdot \varvec{p} \rangle \big )\,\mathrm {d}t. \end{aligned}$$

Secondly, by Galerkin orthogonality we find that

3.35 $$\begin{aligned}&\int _0^T \big (\langle \partial _t e_u , w_\tau \rangle + \langle \nabla \cdot \varvec{e}_{\varvec{q}}, w_\tau \rangle \big )\,\mathrm {d}t = 0, \end{aligned}$$

3.36 $$\begin{aligned}&\int _0^T \big ( \langle \varvec{D}^{-1} \varvec{e}_{\varvec{q}},\varvec{v}_\tau \rangle - \langle e_u, \nabla \cdot \varvec{v}_\tau \rangle \big ) \,\mathrm {d}t = 0 \end{aligned}$$

for all $w_\tau \in \mathcal {Y}^{r-1}(W)$ and $\varvec{v}_\tau \in \mathcal {Y}^{r-1}(\varvec{V})$. Choosing $w_\tau = I_0 z$ in (3.35), it follows that

3.37 $$\begin{aligned} \int _0^T \big (\langle \partial _t e_u , I_0 z \rangle + \langle \nabla \cdot \varvec{e}_{\varvec{q}}, I_0 z \rangle \big )\,\mathrm {d}t = 0. \end{aligned}$$

Further, choosing $\varvec{v}_\tau = \varvec{J}_0 \varvec{p}$ in (3.36) yields that

3.38 $$\begin{aligned} \int _0^T \big ( \langle \varvec{D}^{-1} \varvec{e}_{\varvec{q}},\varvec{J}_0 \varvec{p} \rangle - \langle e_u, \nabla \cdot \varvec{J}_0 \varvec{p}_\tau \rangle \big ) \,\mathrm {d}t = 0 . \end{aligned}$$

Thirdly, combining (3.34) with (3.37) and (3.38), and then using the Cauchy–Schwarz inequality as well as the interpolation error estimates (3.29) and (3.30) yields that

$$\begin{aligned} \Vert e_u \Vert ^2_{L^2(I;W)}&= \int _0^T \big (\langle \partial _t e_u , z-I_0 z\rangle + \langle \nabla \cdot \varvec{e}_q, z-I_0 z \rangle \big )\,\mathrm {d}t \\&\quad - \int _0^T \big (\langle \varvec{D}^{-1} \varvec{e}_{\varvec{q}}, \varvec{p} - \varvec{J}_0 \varvec{p} \rangle - \langle e_u, \nabla \cdot (\varvec{p} - \varvec{J}_0 \varvec{p}) \rangle \big )\,\mathrm {d}t.\\&\le \big (\Vert \partial _t e_u\Vert _{L^2(I;W)} + \Vert \varvec{e}_{\varvec{q}}\Vert _{L^2(I;\varvec{V})} \big )\Vert z-I_0 z\Vert _{L^2(I;W)}\\&\quad + \big (\Vert \varvec{D}^{-1} \Vert _2 \Vert \varvec{e}_{\varvec{q}}\Vert _{_{L^2(I;\varvec{V})}}+ \Vert e_u \Vert _{L^2(I;W)}\big ) \Vert \varvec{p} - \varvec{J}_0 \varvec{p})\Vert _{L^2(I;\varvec{V})}\\&\le \tau \big (\theta _M \Vert \partial _t e_u\Vert _{L^2(I;W)} + \Vert \varvec{e}_{\varvec{q}}\Vert _{L^2(I;\varvec{V})}\big ) \Vert \partial _t z \Vert _{L^2(I;W)} \\&\quad + c \tau \big (\Vert \varvec{e}_{\varvec{q}}\Vert _{L^2(I;\varvec{V})} + \Vert e_u \Vert _{L^2(I;W)}\big ) \Vert \partial _t \varvec{p} \Vert _{L^2(I;\varvec{V})}. \end{aligned}$$

Applying the a priori estimate (3.28) and the additional regularity assumption (3.25) with $g=e_u$ as well as using the error estimate of Theorem 3.5, we then find that

$$\begin{aligned} \Vert e_u \Vert _{L^2(I;W)}&\le c\, \tau \, \left\{ \sum _{n=1}^N \tau _n^{2r} \Big (\Vert \partial _t^{r+1} u\Vert ^2_{L^2(I_n;W)} + \Vert \partial _t^{r+1} \varvec{q} \Vert ^2_{L^2(I_n;\varvec{V})}\Big ) \right\} ^{1/2}\\&\le c\, \tau ^{r+1} \Big (\Vert \partial _t^{r+1} u\Vert _{L^2(I;W)}+\Vert \partial _t^{r+1} \varvec{q} \Vert _{L^2(I;\varvec{V})}\Big ). \end{aligned}$$

This proves the assertion of the theorem. $\square$

Next we derive an error estimate for the non-exact form (2.19), (2.20) of the cGP(*r*) method. The difference of the non-exact form of cGP(*r*) to (2.14), (2.15) comes through the numerically integrated right-hand side term in (2.19). Firstly, we ensure the existence and uniqueness of the solution to the non-exact form of cGP(*r*).

#### Theorem 3.9

(Existence and uniqueness) Let the assumptions of Sect. 2.2 about $\Omega , u_0, \varvec{D}$ and *f* be satisfied. Then the non-exact form (2.19), (2.20) of the semidiscrete problem admits a unique solution $\{U_n^j,\varvec{Q}_n^j\}\in W\times \varvec{V}$ for $j=1,\ldots ,r$ and $n=1,\ldots ,N$ defining semidiscrete approximations $\{u_\tau ,\varvec{q}_\tau \}\in \mathcal {X}^{r}(W)\times \mathcal {X}^{r}(\varvec{V})$ by means of the expansions (2.18) and the initial condition $u_\tau (0)=u_0$.

#### Proof

By the definition of the Lagrange interpolation operator $\Pi _r$ given in (2.21) and the representations (2.18) of $u_\tau$ and $\varvec{q}_\tau$ in terms of basis functions we recast the non-exact form (2.19), (2.20) of the semidiscrete problem in the equivalent form

3.39 $$\begin{aligned} \int _0^T \langle \partial _t u_\tau ,w_\tau \rangle \,\mathrm {d}t + \int _0^T \langle \nabla \cdot \varvec{q}_\tau , w_\tau \rangle \,\mathrm {d}t&= \int _0^T \langle \Pi _r f, w_\tau \rangle \,\mathrm {d}t, \end{aligned}$$

3.40 $$\begin{aligned} \int _0^T \langle \varvec{D}^{-1} \varvec{q}_\tau , \varvec{v}_\tau \rangle \,\mathrm {d}t - \int _0^T \langle u_\tau , \nabla \cdot \varvec{v}_\tau \rangle \,\mathrm {d}t&= 0 \end{aligned}$$

for all $w_\tau \in \mathcal {Y}^{r-1}(W)$ and $\varvec{v} \in \mathcal {Y}^{r-1}(\varvec{V})$ with the initial condition $u_\tau (0) = u_0$.

Existence and uniqueness of the solution $\{u_\tau ,\varvec{q}_\tau \}\in \mathcal {X}^r(W)\times \mathcal {X}^r(\varvec{V})$ to the system (3.39), (3.40) then follows as in Theorems 3.1 and 3.2 with $\Pi _r f$ replacing *f* in the arguments of the proofs. $\square$

Next, we present the corresponding a priori error estimate.

#### Theorem 3.10

Let the assumptions of Sect. 2.2 about $\Omega , u_0, \varvec{D}$ and *f* be satisfied. Suppose that *f* is sufficiently regular with respect to the time variable. Let $\{u,\varvec{q}\} \in H^1(I;W)\times L^2(I;\varvec{V})$ denote the unique solution of the mixed problem (2.10), (2.11) that is supposed to be sufficiently regular. Then the solution $\{u_\tau ,\varvec{q}_\tau \}\in \mathcal {X}^{r}(W)\times \mathcal {X}^{r}(\varvec{V})$ of the non-exact semidiscrete problem (2.14), (2.15) satisfies the error estimatewhere the constants *c* is independent of $\tau _n$, $\tau$ and *T*.

Since the proof of Theorem 3.10 follows from the proof of Theorem 3.5 by a standard estimate of the interpolation error, we skip it here. For the sake of completeness we summarize the proof in the “Appendix” of this work.

## Existence and uniqueness of the fully discrete approximation and error estimates

In the first subsection of Sect. 4 we prove the existence and uniqueness of solutions to the fully discrete approximation scheme (2.28), (2.29). Then, in Sect. 4.2 we establish an estimate for the error between the non-exact form of the semidiscrete approximation defined by Eqs. (2.19), (2.20) and the fully discrete solution given by Eqs. (2.28), (2.29). Finally, in Sect. 4.3 we combine the error estimates of the temporal discretization that are derived in Sect. 3 with the error estimates of Sect. 4.2 to get the desired error estimates.

### Existence and uniqueness of the fully discrete approximation

Firstly we prove the existence and uniqueness of solutions to the fully discrete cGP(*r*)–MFEM(*p*) scheme (2.28), (2.29). For this we need the following lemma (cf. [44, p. 302].

#### Lemma 4.1

For given $w_h \in W_h$ there exits a function $\varvec{v}_h \in \varvec{V}_h$ satisfying

$$\begin{aligned} \nabla \cdot \varvec{v}_h = w_h \quad { \text { and } }\quad \Vert \varvec{v}_h \Vert \le c \Vert w_h \Vert \end{aligned}$$

for some constant $c > 0$ depending on $\Omega$ and the space dimension *d* but not on $w_h$ or the mesh size *h*.

#### Theorem 4.2

(Existence and uniqueness of solutions) Let the assumptions of Sect. 2.2 about $\Omega , u_0, \varvec{D}$ and *f* be satisfied. Then the fully discrete problem (2.28), (2.29) admits a unique solution $\{u_{\tau ,h},\varvec{q}_{\tau ,h}\}\in \mathcal {X}^{r}(W_h)\times \mathcal {X}^{r}(\varvec{V}_h)$.

#### Proof

Since the fully discrete problem (2.28), (2.29) is finite dimensional and linear, it is sufficient to show the uniqueness of the solution. The existence is then a direct consequence. Assume that there exist two pairs of solutions $\{u_{\tau ,h}^k,\varvec{q}_{\tau ,h}^k\}\in \mathcal {X}^{r}(W_h)\times \mathcal {X}^{r}(\varvec{V}_h)$, for $k=1,2$, that are represented in terms of basis functions by

$$\begin{aligned} u_{\tau ,h}^{k} (t)_{|I_n} = \sum _{j=0}^r U_{n,h}^{j,k} \varphi _{n,j}(t) \quad \text{ and } \quad \varvec{q}_{\tau ,h}^{k} (t)_{|I_n} = \sum _{j=0}^r \varvec{Q}_{n,h}^{j,k} \varphi _{n,j}(t), \quad \text{ for }\; k = 1,2, \end{aligned}$$

and $t \in I_n$ with coefficient functions $U_{n,h}^{j,k}\in W_h$ and $\varvec{Q}_{n,h}^{j,k} \in \varvec{V}_h$. The continuity constraint that is imposed by the definition of $\mathcal {X}^{r}(W_h)$ and $\mathcal {X}^{r}(\varvec{V}_h)$, respectively, directly implies that $U_{n,h}^{0,1} = U_{n,h}^{0,2}$ and $\varvec{Q}_{n,h}^{0,1} = \varvec{Q}_{n,h}^{0,2}$. Further, the pairs $\{u_{\tau ,h}^{k} (t), \varvec{q}_{\tau ,h}^{k} (t)\}$, for $k = 1,2$, both satisfy the discrete equations (2.28), (2.29). Therefore, it follows that

4.1 $$\begin{aligned}&\sum _{j=0}^r \hat{\alpha }_{ij} \langle U_{n,h}^{j,1}- U_{n,h}^{j,2} , w_h\rangle + {\tau _n} \, \hat{\beta }_{ii} \langle \nabla \cdot (\varvec{Q}_{n,h}^{i,1}- \varvec{Q}_{n,h}^{i,2}) ,w_h\rangle = 0, \end{aligned}$$

4.2 $$\begin{aligned}&\langle \varvec{D}^{-1} (\varvec{Q}_{n,h}^{i,1}- \varvec{Q}_{n,h}^{i,2}),\varvec{v}_h \rangle - \langle U_{n,h}^{i,1}-U_{n,h}^{i,2},\nabla \cdot \varvec{v}_h\rangle = 0 \end{aligned}$$

for $i=1,\ldots , r$ and all $\{w_h,\varvec{v}_h\}\in W_h\times \varvec{V}_h$. Now, by subtracting the equations (4.1) and (4.2) from each other and choosing the test functions $w_h= U_{n,h}^{i,1} - U_{n,h}^{i,2}$ and $\varvec{v}_h =\tau _n \hat{\beta }_{ii} (\varvec{Q}_{n,h}^{i,1} - \varvec{Q}_{n,h}^{i,2})$, for $i = 1,\ldots , r$ in (4.1) and (4.2), respectively, we get that

4.3 $$\begin{aligned} \sum _{j=0}^r \hat{\alpha }_{ij} \langle U_{n,h}^{j,1} - U_{n,h}^{j,2}, U_{n,h}^{i,1} - U_{n,h}^{i,2}\rangle + {\tau _n} \, \hat{\beta }_{ii} \langle \varvec{D}^{-1} (\varvec{Q}_{n,h}^{i,1} - \varvec{Q}_{n,h}^{i,2}),\varvec{Q}_{n,h}^{i,1} - \varvec{Q}_{n,h}^{i,2} \rangle = 0, \end{aligned}$$

for $i=1,\ldots , r$. Summing up Eq. (4.3) from $i = 1$ to $i=r$, using Lemma 2.4 and recalling that $U_{n,h}^{0,1} = U_{n,h}^{0,2}$ then implies that

4.4 $$\begin{aligned} \dfrac{1}{2} \Vert u_{\tau ,h}^{1} (t_n) - u_{\tau ,h}^{2} (t_n) \Vert ^2 + \sum _{i = 1}^r {\tau _n} \, \hat{\beta }_{ii} \langle \varvec{D}^{-1} (\varvec{Q}_{n,h}^{i,1} - \varvec{Q}_{n,h}^{i,2}),\varvec{Q}_{n,h}^{i,1} - \varvec{Q}_{n,h}^{i,2} \rangle = 0. \end{aligned}$$

The symmetric matrix $\varvec{D}^{-1}$ is positive definite by assumption (2.2) and $\hat{\beta }_{ii} > 0$ under the coefficient property (C); cf. Lemma 2.3. Therefore, Eq. (4.4) immediately implies that $\varvec{Q}_{n,h}^{i,1} = \varvec{Q}_{n,h}^{i,2}$ for $i = 1, \dots , r$. By Lemma 4.1 there exists some $\varvec{v}_h \in \varvec{V}_h$ such that $\nabla \cdot \varvec{v}_h = U_{n,h}^{i,1}-U_{n,h}^{i,2}$. Using this $\varvec{v}_h$ as test function in (4.2) and noting that the first term in (4.2) now vanishes, we obtain that $U_{n,h}^{i,1} = U_{n,h}^{i,2}$, for $i = 1, \dots , r$. This implies the uniqueness of the solution to the fully discrete problem (2.28), (2.29) and proves the assertion of the theorem. $\square$

### Estimates for the error between the semidiscrete and the fully discrete solution

In this subsection we derive estimates for the error between the semidiscrete approximation defined by Eqs. (2.14), (2.15) and the fully discrete solution given by Eqs. (2.28), (2.29). For this we use the following projection operators (cf. [5, 17] and [40, p. 237]) defined in *W* and $\varvec{V}$, respectively, by

4.5 $$\begin{aligned} P_h{:}\,W \rightarrow W_h, \quad \langle P_h w - w, w_h \rangle = 0 \end{aligned}$$

for all $w_h \in W_h$ and

4.6 $$\begin{aligned}&\varvec{\Pi }_h{:}\,\varvec{V} \rightarrow \varvec{V}_h, \quad \langle \nabla \cdot (\varvec{\Pi }_h \varvec{v} - \varvec{v}) , w_h \rangle = 0, \end{aligned}$$

4.7 $$\begin{aligned}&\varvec{P}_h{:}\, \varvec{V} \rightarrow \varvec{V}_h \quad \langle \varvec{P}_h \varvec{v} - \varvec{v}, \varvec{v}_h \rangle = 0, \end{aligned}$$

for all $w_h\in W_h$ and $\varvec{v}_h \in \varvec{V}_h$, respectively. We point out that $\varvec{\Pi }_h$ is firstly defined on $\varvec{H}^1(\Omega )$ and then extended to $\varvec{V}$ by following [40, p. 237]. For these operators and the family of Raviart–Thomas elements on quadrilateral elements for the two-dimensional case and the class of Raviart–Thomas–Nédélec elements in three space dimensions there holds that

4.8 $$\begin{aligned}&\Vert w - P_h w \Vert \le c h^{p+1} \Vert w \Vert _{p+1}, \end{aligned}$$

4.9 $$\begin{aligned}&\Vert \varvec{v} - \varvec{\Pi }_h \varvec{v} \Vert \le c h^{p+1} \Vert \varvec{v} \Vert _{p+1}, \quad \Vert \nabla \cdot (\varvec{v} - \varvec{\Pi }_h \varvec{v}) \Vert \le c h^{p+1} \Vert \nabla \cdot \varvec{v} \Vert _{p+1}, \end{aligned}$$

4.10 $$\begin{aligned}&\Vert \varvec{v} - \varvec{P}_h \varvec{v} \Vert \le c h^{p+1} \Vert \varvec{v} \Vert _{p+1}, \quad \Vert \nabla \cdot (\varvec{v} - \varvec{P}_h \varvec{v}) \Vert \le c h^{p+1} \Vert \nabla \cdot \varvec{v} \Vert _{p+1},\qquad \end{aligned}$$

for any $w \in H^{p + 1}(\Omega )$ and $\varvec{v} \in \varvec{H}^{p+1}(\Omega )$, $\nabla \cdot \varvec{v} \in H^{p+1}(\Omega )$, respectively.

For the error between the semidiscrete solution and fully discrete we use the notation

$$\begin{aligned} E_{u}(t)= & {} u_\tau (t) - u_{\tau ,h} (t), \quad \varvec{E}_{\varvec{q}}(t) = \varvec{q}_\tau (t) - \varvec{q}_{\tau ,h}(t),\\ E_{u,n}^i= & {} E_u(t_{n,i}), \quad \varvec{E}_{\varvec{q},n}^i = \varvec{E}_{\varvec{q}}(t_{n,i}) \end{aligned}$$

for $t\in I$ and $n \in \{1,\ldots ,N\}$, $i \in \{0,\ldots ,r\}$. Representing the semidiscrete and fully discrete solution in terms of basis functions [cf. (2.18)] there holds that

$$\begin{aligned} E_{u}(t)=\sum _{i=0}^r E_{u,n}^i \varphi _{n,i}(t) \quad \text{ and } \quad \varvec{E}_{\varvec{q}}(t)=\sum _{i=0}^r \varvec{E}_{\varvec{q},n}^i \varphi _{n,i}(t), \quad \text{ for } \; t\in I_n. \end{aligned}$$

Next, we prove two preliminary lemmas.

#### Lemma 4.3

Let the assumptions of Sect. 2.2 about $\Omega , u_0, \varvec{D}$ and *f* be satisfied. Let the semidiscrete approximation $\{u_{\tau },\varvec{q}_{\tau }\}\in \mathcal {X}^{r}(W)\times \mathcal {X}^{r}(\varvec{V})$ be defined by (2.18)–(2.20). Further, let $\{u_{\tau ,h},\varvec{q}_{\tau ,h}\}\in \mathcal {X}^{r}(W_h)\times \mathcal {X}^{r}(\varvec{V}_h)$ be the unique solution of the fully discrete problem (2.28), (2.29). Then, for any $K =1,\ldots , N$ it holds that

4.11 $$\begin{aligned}&\displaystyle \Vert E_{u} (t_K) \Vert ^2 + \sum _{n=1}^K \sum _{i=1}^r {\tau _n}\Vert E_{u, n}^i \Vert ^2 + \sum _{n=1}^K \sum _{i=1}^r {\tau _n} \Vert \varvec{E}_{\varvec{q}, n}^i \Vert ^2 \nonumber \\&\displaystyle \quad \le \Vert u_\tau (t_K) - P_h u_\tau (t_K) \Vert ^2 + c \sum _{n=1}^K \sum _{i=1}^r {\tau _n} \left( \Vert U_{n}^i - P_h U_{n}^i \Vert ^2 + \Vert \varvec{Q}_{n}^i - \varvec{\Pi }_h \varvec{Q}_{n}^i \Vert ^2\right) \nonumber \\ \end{aligned}$$

with some constant $c > 0$ not depending on the discretization parameters *h* and $\tau$.

#### Proof

By subtracting (2.28), (2.29) from (2.19), (2.20), respectively, it follows that

4.12 $$\begin{aligned}&\sum _{j=0}^r \hat{\alpha }_{ij} \langle U_{n}^j - U_{n,h}^j, w_h\rangle + {\tau _n} \, \hat{\beta }_{ii} \langle \nabla \cdot (\varvec{Q}_{n}^i - \varvec{Q}_{n,h}^i),w_h\rangle = 0, \end{aligned}$$

4.13 $$\begin{aligned}&\langle \varvec{D}^{-1} (\varvec{Q}_{n}^i - \varvec{Q}_{n,h}^i),\varvec{v}_h \rangle - \langle U_{n}^i - U_{n,h}^i,\nabla \cdot \varvec{v}_h\rangle = 0 \end{aligned}$$

for $$i=1,\ldots , r$$ and all $$\{w_h,\varvec{v}_h\}\in W_h\times \varvec{V}_h$$. For any $$i=1,\ldots , r$$ we choose the test functions $$w_h = P_h U_{n}^i - U_{n,h}^i \in W_h$$ and $$\varvec{v}_h = {\tau _n} \, \hat{\beta }_{ii} (\varvec{\Pi }_h \varvec{Q}_{n}^i - \varvec{Q}_{n,h}^i) \in \varvec{V}_h$$ in (4.12) and (4.13), respectively. By adding the thus obtained equations, using the properties of the projection projectors $$P_h$$ and $$\varvec{\Pi }_h$$ defined in (4.5) and (4.6), respectively, and summing up from $$i = 1 $$ to *r* we get that

4.14 $$\begin{aligned}&\sum _{i=1}^r \sum _{j=0}^r \hat{\alpha }_{ij} \langle P_h U_{n}^j - U_{n,h}^j, P_h U_{n}^i - U_{n,h}^i \rangle \nonumber \\&\quad + \sum _{i=1}^r {\tau _n} \, \hat{\beta }_{ii} \langle \varvec{D}^{-1} (\varvec{Q}_{n}^i - \varvec{Q}_{n,h}^i), \varvec{\Pi }_h \varvec{Q}_{n}^i - \varvec{Q}_{n,h}^i \rangle = 0. \end{aligned}$$

We note that due to Lemma 2.4, the first term in (4.14) can be rewritten as

$$\begin{aligned}&\sum _{i=1}^r \sum _{j=0}^r \hat{\alpha }_{ij} \langle P_h U_{n}^j - U_{n,h}^j, P_h U_{n}^i - U_{n,h}^i \rangle \\&\quad = \dfrac{1}{2}\Vert P_h E_{u,n} (t_n) \Vert ^2 - \dfrac{1}{2}\Vert P_h E_{u,n-1} (t_{n-1}) \Vert ^2. \end{aligned}$$

Along with some further algebraic manipulations we then conclude from (4.14) that

4.15 $$\begin{aligned}&\dfrac{1}{2}\Vert P_h E_{u,n} (t_n) \Vert ^2 - \dfrac{1}{2}\Vert P_h E_{u,n-1} (t_{n-1}) \Vert ^2 \nonumber \\&\quad + \sum _{i=1}^r {\tau _n} \, \hat{\beta }_{ii} \left\langle \varvec{D}^{-1} (\varvec{Q}_{n}^i - \varvec{Q}_{n,h}^i), \varvec{Q}_{n}^i - \varvec{Q}_{n,h}^i \right\rangle \nonumber \\&\quad = \sum _{i=1}^r {\tau _n} \, \hat{\beta }_{ii} \left\langle \varvec{D}^{-1} (\varvec{Q}_{n}^i - \varvec{Q}_{n,h}^i), \varvec{Q}_{n}^i - \varvec{\Pi }_h \varvec{Q}_{n}^i \right\rangle . \end{aligned}$$

Recalling assumption (2.2) about $\varvec{D}$ and property (C) in (2.22) of the coefficients $\hat{\beta }_{ii}$ and we obtain from Eq. (4.15) by applying Cauchy–Young’s inequality that

4.16 $$\begin{aligned}&\displaystyle \Vert P_h E_{u} (t_n) \Vert ^2 - \Vert P_h E_{u} (t_{n-1}) \Vert ^2 + \sum _{i=1}^r {\tau _n} \beta _m \theta _m \Vert \varvec{Q}_{n}^i - \varvec{Q}_{n,h}^i\Vert ^2 \nonumber \\&\quad \le \frac{\beta _M^2}{\beta _m \, \theta _m} \sum _{i=1}^r {\tau _n} \Vert \varvec{Q}_{n}^i - \varvec{\Pi }_h \varvec{Q}_{n}^i \Vert ^2. \end{aligned}$$

Summing up inequality (4.16) from $n = 1$ to *K* and noting that $P_h E_{u}(t_{0}) = 0$ then shows that

4.17 $$\begin{aligned}&\Vert P_h E_{u} (t_K) \Vert ^2 + \sum _{n=1}^K \sum _{i=1}^r {\tau _n} \beta _m \Vert \varvec{Q}_{n}^i - \varvec{Q}_{n,h}^i\Vert ^2 \nonumber \\&\quad \le \frac{\beta _M^2}{\beta _m \, \theta _m} \sum _{n=1}^K \sum _{i=1}^r {\tau _n} \Vert \varvec{Q}_{n}^i - \varvec{\Pi }_h \varvec{Q}_{n}^i \Vert ^2 \end{aligned}$$

for any $K \in \mathbb {N}$ with $K \le N$. By using now Lemma 4.1, there exists for any $i \in \{1, \ldots , r\}$ a $\varvec{v}_h \in \varvec{V}_h$ such that $\nabla \cdot \varvec{v}_h = P_h E_{u,n}^i$ and $\Vert \varvec{v}_h \Vert \le c \Vert P_h E_{u,n}^i \Vert$. By testing (4.13) with this $\varvec{v}_h$, we get by using the Cauchy–Schwarz inequality along with assumption (2.2) about $\varvec{D}$ that

4.18 $$\begin{aligned} \Vert P_h E_{u,n}^i \Vert \le c \, \theta _M \Vert \varvec{Q}_{n}^i - \varvec{Q}_{n,h}^i \Vert , \end{aligned}$$

for $$n=1,\ldots , N$$, $$i=1,\ldots , r$$.

Combining (4.17) with (4.18) is follows that

4.19 $$\begin{aligned}&\Vert P_h E_{u} (t_K) \Vert ^2 + \sum _{n=1}^K \sum _{i=1}^r {\tau _n} \beta _m \Vert \varvec{Q}_{n}^i - \varvec{Q}_{n,h}^i\Vert ^2 + \sum _{n=1}^K \sum _{i=1}^r \tau _n \Vert P_h E_{u,n}^i \Vert ^2 \nonumber \\&\quad \le c \sum _{n=1}^K \sum _{i=1}^r \tau _n \Vert \varvec{Q}_{n}^i - \varvec{\Pi }_h \varvec{Q}_{n}^i \Vert ^2. \end{aligned}$$

By $\varvec{E}_{\varvec{q},n}^{i} = \varvec{Q}_{n}^i - \varvec{Q}_{n,h}^i$ and the triangle inequality relation (4.19) implies that

4.20 $$\begin{aligned} \begin{aligned} \Vert&E_{u} (t_K) \Vert ^2 + \sum _{n=1}^K \sum _{i=1}^r {\tau _n} \beta _m \Vert \varvec{E}_{\varvec{q},n}^{i} \Vert ^2 + \sum _{n=1}^K \sum _{i=1}^r \tau _n \Vert E_{u,n}^i \Vert ^2 \\&\le c \sum _{n=1}^K \sum _{i=1}^r \tau _n \Vert \varvec{Q}_{n}^i - \varvec{\Pi }_h \varvec{Q}_{n}^i \Vert ^2 + c \Vert u_{\tau }(t_k) - P_h u_{\tau }(t_k)\Vert ^2\\&\quad + c \sum _{n=1}^K \sum _{i=1}^r \tau _n \Vert E_{u,n}^i - P_h E_{u,n}^i\Vert ^2. \end{aligned} \end{aligned}$$

Observing that $E_{u,n}^i - P_h E_{u,n}^i = U_n^i - P_h U_n^i$, inequality (4.20) proves (4.11). $\square$

In the second lemma we restrict ourselves to the case that $\varvec{D} = d\varvec{I}$ with some $d>0$ is satisfied. An extension of the provided estimates to more general matrices $\varvec{D}(\varvec{x})$ still remains an open problem.

#### Lemma 4.4

Let the assumptions of Sect. 2.2 about $\Omega , u_0$ and *f* be satisfied and $\varvec{D} = d\varvec{I}$ with some $d>0$. Let the semidiscrete approximation $\{u_{\tau },\varvec{q}_{\tau }\}\in \mathcal {X}^{r}(W)\times \mathcal {X}^{r}(\varvec{V})$ be defined by (2.18)–(2.20). Further, let $\{u_{\tau ,h},\varvec{q}_{\tau ,h}\}\in \mathcal {X}^{r}(W_h)\times \mathcal {X}^{r}(\varvec{V}_h)$ be the unique solution of the fully discrete problem (2.28), (2.29). Then, for any $K =1,\ldots , N$ it holds that

4.21 $$\begin{aligned}&\sum _{n=1}^K \tau _n \sum _{i=1}^r \hat{\beta }_{ii}\Vert \nabla \cdot \varvec{\Pi }_h \varvec{E}_{\varvec{q},n}^i \Vert ^2 + \Vert \varvec{P}_h \varvec{E}_{\varvec{q}} (t_K) \Vert ^2 \nonumber \\&\quad \le \sum _{n=1}^K \sum _{i=1}^r \tau _n \, \hat{\beta }_{ii} \Vert \nabla \cdot \varvec{(}\varvec{P}_h - \varvec{\Pi }_h) \varvec{Q}_{n}^i\Vert ^2. \end{aligned}$$

#### Proof

Introducing the projectors into the error equations (4.12)–(4.13) yields that

4.22 $$\begin{aligned}&\sum _{j=0}^r \hat{\alpha }_{ij} \langle P_h E_{u,n}^j, w_h\rangle + {\tau _n} \, \hat{\beta }_{ii} \langle \nabla \cdot \varvec{\Pi }_h \varvec{E}_{\varvec{q},n}^i,w_h\rangle = 0, \end{aligned}$$

4.23 $$\begin{aligned}&\langle \varvec{P}_h \varvec{E}_{\varvec{q},n}^i, \varvec{v}_h \rangle - \langle P_h E_{u,n}^i,\nabla \cdot \varvec{v}_h\rangle = 0 \end{aligned}$$

for $n=1,\ldots , N$, $i=1,\ldots , r$ and all $\{w_h,\varvec{v}_h\}\in W_h\times \varvec{V}_h$. Observing that for any $n \ge 2$ the quantities $\varvec{E}_{\varvec{q},n}^0$ and $E_{u,n}^0$ are linear combinations of $\varvec{E}_{\varvec{q},n-1}^i$ and $\varvec{E}_{u,n-1}^i$, for $i = 0, \dots , r$, respectively, and that $P_h E_{u,1}^0 = 0$ and $\varvec{P}_h \varvec{E}_{\varvec{q},1}^0 = \varvec{0}$ by definition of $\{U_1^0, \varvec{Q}_1^0\}$ and $\{U_{n,h}^1,\varvec{Q}_{n,h}^1\}$, it follows that Eq. (4.23) is also satisfied for $i = 0$ and any $n \ge 1$. Using this, we obtain by multiplying (4.23) with $\hat{\alpha }_{ji}$ and summing up the resulting identity from $i = 0$ to *r* that

4.24 $$\begin{aligned} \left\langle \sum _{j = 0}^r \hat{\alpha }_{ij} \varvec{P}_h \varvec{E}_{\varvec{q},n}^j, \varvec{v}_h \right\rangle - \left\langle \sum _{j = 0}^r \hat{\alpha }_{ij} P_h E_{u,n}^j,\nabla \cdot \varvec{v}_h\right\rangle = 0 \end{aligned}$$

for any $\varvec{v}_h \in \varvec{V}_h$. We note that we changed the notation for the indices. By testing now (4.22) with $w_h = \sum _{j = 0}^r \hat{\alpha }_{ij} P_h E_{u,n}^j \in W_h$ and (4.24) with $\varvec{v}_h = \tau _n \, \hat{\beta }_{ii} \varvec{P}_h \varvec{E}_{\varvec{q},n}^i \in \varvec{V}_h$, we get by summing the resulting equations and using the inequalities of Cauchy–Schwarz and Cauchy–Young that

$$\begin{aligned}&\Big \Vert \sum _{j = 0}^r \hat{\alpha }_{ij} P_h E_{u,n}^j \Big \Vert ^2 + \; \tau _n \, \hat{\beta }_{ii} \Big \langle \sum _{j = 0}^r \hat{\alpha }_{ij} \varvec{P}_h \varvec{E}_{\varvec{q},n}^j, \varvec{P}_h \varvec{E}_{\varvec{q},n}^i \Big \rangle \\&\quad = \; \tau _n \, \hat{\beta }_{ii} \Big \langle \sum _{j = 0}^r \hat{\alpha }_{ij} P_h E_{u,n}^j, \nabla \cdot \varvec{(}\varvec{P}_h - \varvec{\Pi }_h) \varvec{E}_{\varvec{q},n}^i \Big \rangle \\&\quad \le \; \dfrac{1}{2} \Big \Vert \sum _{j = 0}^r \hat{\alpha }_{ij} P_h E_{u,n}^j \Big \Vert ^2 + \dfrac{1}{2} \tau _n^2 \, \hat{\beta }_{ii}^2 \Vert \nabla \cdot (\varvec{P}_h - \varvec{\Pi }_h) \varvec{E}_{\varvec{q},n}^i\Vert ^2 \end{aligned}$$

for $n=1,\ldots , N$ and $i=1,\ldots , r$. The inequality above further simplifies to

4.25 $$\begin{aligned}&\Big \Vert \sum _{j = 0}^r \hat{\alpha }_{ij} P_h E_{u,n}^j \Big \Vert ^2 + 2 \tau _n \, \hat{\beta }_{ii} \Big \langle \sum _{j = 0}^r \hat{\alpha }_{ij} \varvec{P}_h \varvec{E}_{\varvec{q},n}^j, \varvec{P}_h \varvec{E}_{\varvec{q},n}^i \Big \rangle \nonumber \\&\quad \le \displaystyle \tau _n^2 \, \hat{\beta }_{ii}^2 \Vert \nabla \cdot (\varvec{P}_h - \varvec{\Pi }_h) \varvec{E}_{\varvec{q},n}^i\Vert ^2, \end{aligned}$$

for $n=1,\ldots , N$, $i=1,\ldots , r$. Dividing (4.25) by $\tau _n \,\hat{\beta }_{ii}$ (note that $\hat{\beta }_{ii} > 0$ for all $i =1, \ldots , r$), summing up the resulting inequality from $i=1,\ldots , r$ and using Lemma 2.4 gives that

4.26 $$\begin{aligned}&\sum _{i=1}^r \dfrac{1}{ \tau _n \, \hat{\beta }_{ii} } \Big \Vert \sum _{j = 0}^r \hat{\alpha }_{ij} P_h E_{u,n}^j \Big \Vert ^2 + \Vert \varvec{P}_h \varvec{E}_{\varvec{q}} (t_n) \Vert ^2 \le \displaystyle \Vert \varvec{P}_h \varvec{E}_{\varvec{q}} (t_{n-1}) \Vert ^2 \nonumber \\&\quad +\,\sum _{i=1}^r \tau _n \, \hat{\beta }_{ii} \Vert \nabla \cdot (\varvec{P}_h - \varvec{\Pi }_h) \varvec{E}_{\varvec{q},n}^i\Vert ^2 \end{aligned}$$

for $n=1,\ldots , N$. By summing up (4.26) from $n=1,\ldots , K$ and noting that $\varvec{P}_h \varvec{E}_{\varvec{q}} (t_{0}) = \varvec{P}_h \varvec{E}_{\varvec{q},1}^0=\varvec{0}$ for the choices of the semidiscrete and fully discrete coefficient functions $\varvec{Q}_{1}^0$ and $\varvec{Q}_{1,h}^0$ [cf. their definition below (2.19), (2.20) and Eq. (2.30)] we get that

4.27 $$\begin{aligned}&\sum _{n=1}^K \sum _{i=1}^r \dfrac{1}{ \tau _n \, \hat{\beta }_{ii} } \Big \Vert \sum _{j = 0}^r \hat{\alpha }_{ij} P_h E_{u,n}^j \Big \Vert ^2 + \Vert \varvec{P}_h \varvec{E}_{\varvec{q}} (t_K) \Vert ^2 \nonumber \\&\quad \le \sum _{n=1}^K \sum _{i=1}^r \tau _n \, \hat{\beta }_{ii} \Vert \nabla \cdot \varvec{(}\varvec{P}_h - \varvec{\Pi }_h) \varvec{E}_{\varvec{q},n}^i\Vert ^2. \end{aligned}$$

We now estimate the divergence of the flux. By testing (4.22) with $w_h = \nabla \cdot \varvec{\Pi }_h \varvec{E}_{\varvec{q},n}^i \in W_h$, and using the inequalities of Cauchy–Schwarz and Cauchy–Young ($\hat{\beta }_{ii} > 0$ for all $i=1, \ldots , r$) we get that

$$\begin{aligned} {\tau _n} \, \hat{\beta }_{ii} \Vert \nabla \cdot \varvec{\Pi }_h \varvec{E}_{\varvec{q},n}^i \Vert ^2&= - \Big \langle \sum _{j=0}^r \hat{\alpha }_{ij} P_h E_{u,n}^j,\nabla \cdot \varvec{\Pi }_h \varvec{E}_{\varvec{q},n}^i \Big \rangle \\&\le \dfrac{1}{2 {\tau _n} \, \hat{\beta }_{ii}} \Big \Vert \sum _{j=0}^r \hat{\alpha }_{ij} P_h E_{u,n}^j\Big \Vert ^2 + \dfrac{{\tau _n} \, \hat{\beta }_{ii}}{2} \Big \Vert \nabla \cdot \varvec{\Pi }_h \varvec{E}_{\varvec{q},n}^i \Big \Vert ^2 \end{aligned}$$

for $n=1,\ldots , N$, $i=1,\ldots , r$. Summing up the previous inequality from $n=1,\ldots , K$ as well as from $i=1,\ldots , r$, using (4.27) along with $(\varvec{P}_h - \varvec{\Pi }_h) \varvec{E}_{\varvec{q},n}^i = (\varvec{P}_h - \varvec{\Pi }_h) \varvec{Q}_{n}^i$ by definition of the projectors $\varvec{P}_h$ and $\varvec{\Pi }_h$ we obtain that

$$\begin{aligned} \sum _{n=1}^K \sum _{i=1}^r {\tau _n} \, \hat{\beta }_{ii} \Vert \nabla \cdot \varvec{\Pi }_h \varvec{E}_{\varvec{q},n}^i \Vert ^2 \le \sum _{n=1}^K \sum _{i=1}^r \tau _n \, \hat{\beta }_{ii} \Vert \nabla \cdot (\varvec{P}_h - \varvec{\Pi }_h) \varvec{Q}_n^i\Vert ^2, \end{aligned}$$

which proves the assertions of the lemma. $\square$

Now we combine the inequalities of the previous lemmas to estimate the error between the semidiscrete and the fully discrete solutions in the norms of $L^2(I; W)$ and $L^2(I; \varvec{V})$.

#### Theorem 4.5

Let the assumptions of Sect. 2.2 about $\Omega , u_0, \varvec{D}$ and *f* be satisfied. Let the sufficiently regular semidiscrete approximation $\{u_{\tau },\varvec{q}_{\tau }\}\in \mathcal {X}^{r}(W)\times \mathcal {X}^{r}(\varvec{V})$ be defined by (2.18)–(2.20). Further, let $\{u_{\tau ,h},\varvec{q}_{\tau ,h}\}\in \mathcal {X}^{r}(W_h)\times \mathcal {X}^{r}(\varvec{V}_h)$ be the solution of the fully discrete problem (2.28), (2.29). For the scalar variable $u_\tau$ it holds that

4.28 $$\begin{aligned} \Vert u_\tau - u_{\tau , h} \Vert _{L^2(I; W)}&\le c h^{p+1} . \end{aligned}$$

For the vectorial variable $\varvec{q}_\tau$ it holds that

4.29 $$\begin{aligned} {\left( \sum _{n=1}^N \tau _n \sum _{i=1}^r \Vert \varvec{q}_\tau (t_{n,i}) - \varvec{q}_{\tau ,h}(t_{n,i}) \Vert ^2\right) ^{1/2} \le c h^{p+1}.} \end{aligned}$$

Further, for $\varvec{D}(\varvec{x}) = d \varvec{I}$, for some $d>0$, it holds that

4.30 $$\begin{aligned} \Vert \varvec{q}_\tau - \varvec{q}_{\tau , h} \Vert _{L^2(I; \varvec{L}^2(\Omega ))}&\le c h^{p+1} \end{aligned}$$

and

4.31 $$\begin{aligned} {\left( \sum _{n=1}^N \tau _n \sum _{i=1}^r \Vert \varvec{q}_\tau (t_{n,i}) - \varvec{q}_{\tau ,h}(t_{n,i}) \Vert _{\varvec{V}}^2\right) ^{1/2} \le c h^{p+1}.} \end{aligned}$$

The constant *c* does not depend on the discretization parameters *h* and $\tau$.

#### Proof

By using (2.25) and recalling that $E_{u,n}^0 = E_u(t_{n-1})$ we find that

4.32 $$\begin{aligned} \Vert u_\tau - u_{\tau , h}\Vert _{L^2(I; W)}^2 \le c \bigg (\sum _{n=1}^N \sum _{i=1}^r \tau _n \Vert E_{u,n}^i \Vert ^2 + \sum _{n=1}^N \tau _n \Vert E_{u}(t_{n-1})\Vert ^2 \bigg ). \end{aligned}$$

By inequality (4.11) we get for the first term on the right-hand side of (4.32) that

4.33 $$\begin{aligned}&\sum _{n=1}^N \sum _{i=1}^r \tau _n \Vert E_{u,n}^i \Vert ^2 { + \sum _{n=1}^N \sum _{i=1}^r \tau _n \Vert \varvec{E}_{\varvec{q},n}^i \Vert ^2 } \le c \bigg (\sum _{n=1}^N \sum _{i=1}^r \tau _n \Vert U_n^i - P_h U_n^i \Vert ^2 \nonumber \\&\quad +\,\tau _N \Vert u_{\tau }(t_N) - P_h u_{\tau }(t_N) \Vert ^2 + \sum _{n=1}^N \sum _{i=1}^r \tau _n \Vert \varvec{Q}_n^i - \varvec{\Pi }_h \varvec{Q}_n^i \Vert ^2\bigg ). \end{aligned}$$

Using (4.11) again, we find for the second term on the right-hand side of (4.32) that

4.34 $$\begin{aligned}&\Vert E_{u}(t_K)\Vert ^2 \le \Vert u_\tau (t_K)-P_h u_\tau (t_k)\Vert ^2 \nonumber \\&\quad +\,c \sum _{n=1}^K \sum _{i=1}^r \left( \Vert U_n^i - P_h U_n^i \Vert ^2 + \Vert \varvec{Q}_n^i - \varvec{\Pi }_h \varvec{Q}_n^i\Vert ^2\right) \end{aligned}$$

for $K=1,\ldots , N$. Combining now (4.32) with (4.33) and (4.34) and using the approximation properties (4.9)–(4.10) of the projection operators we then get that

4.35 $$\begin{aligned}&\Vert u_\tau - u_{\tau , h} \Vert _{L^2(I; W)}^2\le c \bigg (\sum _{n=1}^N \tau _n h^{2(p+1)} \sum _{i=0}^r \Vert U_n^i \Vert _{p+1}^2 \nonumber \\&\quad +\,h^{2(p+1)} \max _{K=0,\ldots , N} \Vert u_{\tau }(t_K) \Vert _{p+1}^2 + \sum _{n=1}^N \tau _n h^{2(p+1)} \sum _{i=0}^r \Vert \varvec{Q}_n^i \Vert _{p+1}^2 \bigg ) , \end{aligned}$$

where the arising constant does not depend on the discretization parameters *h* and $\tau$. The result (4.28) directly follows from (4.35) under the assumption of the theorem of sufficiently regular coefficient functions $\{U_n^j,\varvec{Q}_n^j\}\in W\times \varvec{V}$. From (4.33) along with (4.34) and (4.9)–(4.10) we further conclude that

4.36 $$\begin{aligned} \sum _{n=1}^N \tau _n \sum _{i=1}^r \Vert \varvec{E}_{\varvec{q}}(t_{n,i}) \Vert ^2 \le c h^{2(p+1)}. \end{aligned}$$

This proves (4.29).

By using (2.25), recalling that $\varvec{E}_{\varvec{q},n}^0 = \varvec{E}_{\varvec{q}}(t_{n-1})$ and applying the boundedness of the $\varvec{L}^2$ projection operator $\varvec{P}_h$ we find that

4.37 $$\begin{aligned}&\Vert \varvec{q}_\tau - \varvec{q}_{\tau , h} \Vert _{L^2(I; \varvec{L}^2(\Omega ))}^2 \nonumber \\&\quad \le c \Big (\Vert \varvec{q}_\tau - \varvec{P}_h \varvec{q}_{\tau } \Vert _{L^2(I; \varvec{L}^2(\Omega ))}^2 + \Vert \varvec{P}_h \varvec{q}_\tau - \varvec{q}_{\tau , h} \Vert _{L^2(I; \varvec{L}^2(\Omega ))}^2\Big ) \nonumber \\&\quad \le c \bigg (\sum _{n=1}^N \sum _{i=1}^r \tau _n \Vert \varvec{E}_{\varvec{q},n}^i \Vert ^2 + \sum _{n=1}^N \tau _n \Vert \varvec{P}_h \varvec{E}_{\varvec{q}}(t_{n-1})\Vert ^2 \nonumber \\&\qquad +\,\Vert \varvec{q}_\tau - \varvec{P}_h\varvec{q}_{\tau } \Vert _{L^2(I; \varvec{L}^2(\Omega ))}^2 \bigg ). \end{aligned}$$

For $\varvec{D}(\varvec{x}) = d \varvec{I}$ the second term on the right-hand side of (4.37) can be bounded from above by means of the inequality (4.21) along with the observation that $(\varvec{P}_h - \varvec{\Pi }_h) \varvec{E}_{\varvec{q},n}^i = (\varvec{P}_h - \varvec{\Pi }_h) \varvec{Q}_{n}^i$ by definition of the projectors $\varvec{P}_h$ and $\varvec{\Pi }_h$. Recalling further the boundedness of $\hat{\beta }_{ii}$ (cf. Lemma 2.3) we conclude that

4.38 $$\begin{aligned} \Vert \varvec{P}_h \varvec{E}_{\varvec{q}}(t_K)\Vert ^2 \le c \sum _{n=1}^K \sum _{i=1}^r \tau _n \Vert (\varvec{P}_h - \varvec{\Pi }_h) \varvec{Q}_{n}^i \Vert ^2 \end{aligned}$$

for $K=1,\ldots , N$. Finally, combining (4.37) with (4.33) and (4.38) and using the approximation properties (4.9)–(4.10) of the projection operators we then get that

4.39 $$\begin{aligned}&\Vert \varvec{q}_\tau - \varvec{q}_{\tau , h} \Vert _{L^2(I; \varvec{L}^2(\Omega ))}^2 \nonumber \\&\quad \le \; c \bigg (\sum _{n=1}^N \tau _n h^{2(p+1)} \sum _{i=0}^r \Vert U_n^i \Vert _{p+1}^2 + h^{2(p+1)} \max _{K=0,\ldots , N} \Vert u_{\tau }(t_K) \Vert _{p+1}^2 \nonumber \\&\qquad + \sum _{n=1}^N \tau _n h^{2(p+1)} \sum _{i=0}^r \Vert \varvec{Q}_n^i \Vert _{p+1}^2 { + h^{2(p+1)} \Vert \varvec{q}_\tau \Vert ^2_{L^2\left( I;\varvec{H}^{p+1}(\Omega )\right) }}\bigg ) , \end{aligned}$$

where the arising constant does not depend on the discretization parameters *h* and $\tau$. The result (4.30) directly follows from (4.39) under the assumption of sufficiently regular coefficient functions $\{U_n^j,\varvec{Q}_n^j\}\in W\times \varvec{V}$.

To estimate the divergence part of the error in (4.31), we use that by definition of the projection operators it holds that

4.40 $$\begin{aligned} \begin{aligned}&\sum _{n=1}^N \tau _n \sum _{i=1}^r \Vert \nabla \cdot \varvec{E}_{\varvec{q}}(t_{n,i}) \Vert ^2 \\&\quad \le \sum _{n=1}^N \tau _n \sum _{i=1}^r \Vert \nabla \cdot (\varvec{Q}_{n}^i- \varvec{\Pi }_h \varvec{Q}_{n}^i) \Vert ^2 + \sum _{n=1}^N \tau _n \sum _{i=1}^r \Vert \nabla \cdot \varvec{\Pi }_h \varvec{E}_{\varvec{q},n}^i \Vert ^2 . \end{aligned} \end{aligned}$$

The assertion (4.31) then follows from (4.40) combined with (4.21) and the approximation properties (4.9)–(4.10). $\square$

We remark that the inequalities (4.30) and (4.31) provide an error control for the spatial discretization in the Gaussian quadrature points or temporal degrees of freedom of the subintervals $I_n$ with respect to the norm of $\varvec{L}^2(\Omega )$ and $\varvec{V}$, respectively. For an error control with respect to the norm of $L^2(I;\varvec{V})$ or $L^2(I;\varvec{V})$ a further estimate of $E_{\varvec{q},n}^{0}$ is required which remains an open problem.

### Error estimates for the error between the continuous and the fully discrete solution

In this section we combine the results of Theorems 3.8 and 3.10 with the estimates of Theorem 4.5 to prove the convergence of the fully discrete scheme.

#### Theorem 4.6

Let the assumptions of Sect. 2.2 about $\Omega , u_0, \varvec{D}$ and *f* be satisfied. Let $\{u,\varvec{q}\} \in H^1(I;W)\times L^2(I;\varvec{V})$ denote the unique solution of (2.10), (2.11) that is supposed to be sufficiently regular. Further, let $\{u_{\tau ,h},\varvec{q}_{\tau ,h}\}\in \mathcal {X}^{r}(W_h)\times \mathcal {X}^{r}(\varvec{V}_h)$ be the uniquely defined solution of the fully discrete problem (2.28), (2.29), respectively. Suppose that the semidiscrete problem (2.19), (2.20) admits a sufficiently regular solution $\{u_{\tau },\varvec{q}_{\tau }\}\in \mathcal {X}^{r}(W)\times \mathcal {X}^{r}(\varvec{V})$. Then, there holds that

4.41 $$\begin{aligned} \Vert u - u_{\tau , h} \Vert _{L^2(I; L^2(\Omega ))}&\le c (\tau ^{r} + h^{p+1}). \end{aligned}$$

For homogeneous diffusion coefficients $\varvec{D}(\varvec{x}) = d \varvec{I}$, with some constant $d>0$, there holds that

4.42 $$\begin{aligned} \Vert \varvec{q} - \varvec{q}_{\tau , h} \Vert _{L^2(I; \varvec{L}^2(\Omega ))} \le c (\tau ^{r} + h^{p+1}). \end{aligned}$$

Under the regularity condition (R$_{\mathrm {mix}})$ given in (3.25) and for interpolated right-hand side functions (2.21) there holds that

4.43 $$\begin{aligned} \Vert u - u_{\tau , h} \Vert _{L^2(I; L^2(\Omega ))} \le c (\tau ^{r+1} + h^{p+1}). \end{aligned}$$

The constant *c* in (4.41)–(4.43), respectively, does not depend on the discretization parameters *h* and $\tau$.

#### Proof

By using the triangle inequality, Theorems 3.10 and 4.5 it follows that

$$\begin{aligned} \Vert u - u_{\tau , h} \Vert _{L^2(I; L^2(\Omega ))}^2&\le 2 \Vert u - u_{\tau } \Vert _{L^2(I; L^2(\Omega ))}^2 + 2 \Vert u_{\tau } - u_{\tau , h} \Vert _{L^2(I; L^2(\Omega ))}^2 \\&\le c \left( \tau ^{2r} + h^{2(p+1)}\right) , \end{aligned}$$

where sufficient regularity of the continuous and semidiscrete solution with appropriate upper bounds for the solutions (cf. Theorems 3.10 and 4.5) is assumed. The inequality (4.42) is obtained similarly. The estimate (4.43) can be concluded in the same way by using now the result of Theorem 3.8 instead of Theorem 3.10. $\square$

#### Remark 4.7

- The error estimate (4.43) is optimal in time and space. The assumption of an interpolated right-hand side function (2.21) can still be dropped even though this is not explicitly done in this work. It requires to estimate the error between the exact form of cGP(*r*) defined in (2.14), (2.15) and the fully discrete solution. In this case the arguments used to prove Theorem 4.5 have to be augmented by an estimate of the interpolation error for the right-hand side function, similarly to the proof of Theorem 3.10.
- The error estimate (4.42) is suboptimal in time. It remains an open problem to analyze if the estimates can still be sharpened to order $r+1$. In our numerical study presented in Sect. 5 convergence of order $r+1$ will be observed for the temporal discretization of the scalar and the flux variable. Moreover, this is even observed in the (spatially) stronger norm of $L^2(0,T;\varvec{V})$ instead of $L^2(0,T;\varvec{L}^2(\Omega ))$ for the flux variable.

## Numerical studies

In this section we present numerical studies in order to illustrate the error estimate given in Theorem 4.6 for the fully discrete scheme (2.28), (2.29) combining a variational time discretization with the MFEM. Moreover, we analyze the robustness of the convergence behaviour with respect to random perturbations of the meshes. Thereby we mimic mesh distributions of applications that are of practical interest. Additional convergence studies for variational space-time discretizations of the proposed type as well as for discontinuous time discretizations can be found in [15, 37] for parabolic problems and in [36, 37] for variational space-time discretizations of wave equations. In [15, 37] the efficient iterative solution of the resulting algebraic system of Eqs. (2.28), (2.29) along with the construction of appropriate preconditioning techniques is carefully addressed. In the literature, further computational studies of variational time discretization schemes are presented also for different kind of flow and transport problems in, e.g., [1–3, 30–32, 38, 46].

In order to determine the space-time convergence behavior we consider in our numerical study the cGP(2)–MFEM(2) approach. That is (2.14)–(2.15) with $r=2$ combined with the mixed finite element method MFEM(2) based on the choice $p=2$ in the definition (2.26) and (2.27) of the tuple of MFE spaces. We prescribe the solution

$$\begin{aligned} u_{ E }(\varvec{x}, t) := \sin (\omega t) \sin (\pi x_1) \sin (\pi x_2), \quad in \quad \Omega \times (0,T), \end{aligned}$$

with $\Omega = (0,1)^2$, $\omega = 10\pi$ of problem (2.6)–(2.8). The corresponding flux function is then given by $\varvec{q}_{ E } = -\varvec{D} \nabla u_{ E }$ for $\varvec{D} = \varvec{I}$. We choose the final time $T=1$. On the coarsest level (level 0) the temporal mesh is uniformly refined into $N=10$ time subintervals and the corresponding spatial mesh consists of a single cell. In the following, we use the abbreviation

$$\begin{aligned} e_{u}^{ cGP (2)}(t) := u_{ E }(t) - u_{\tau ,h}(t)\quad and \quad e_{{\varvec{q}}}^{ cGP (2)}(t) := {\varvec{q}}_{ E }(t) - {\varvec{q}}_{\tau ,h}(t), \end{aligned}$$

where we denote by $u_{\tau ,h}$ and by ${\varvec{q}}_{\tau ,h}$ the fully discrete cGP(2)–MFEM(2) approximation of the primal variable and the flux field. The discretization errors for $e_u^{ cGP(2) }$ are measured in the $L^2(I; L^2(\Omega ))$-norm and for $\varvec{e}_{\varvec{q}}^{ cGP(2) }$ in the $L^2(I; \varvec{V})$-norm. As usual, the integral over the spatial domain $\Omega$ and the integral over the time domain $I=(0,T)$ in the error norms are evaluated elementwise in space and time by appropriate quadrature rules of sufficiently high order of accuracy.

### Uniform meshes

**Fig. 1:**
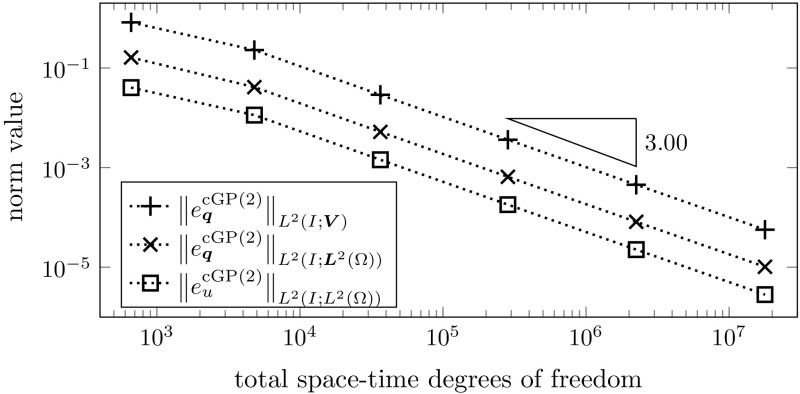
Calculated errors and experimental orders of convergence in space-time for cGP(2)–MFEM(2)

To determine the experimental orders of convergence the space-time mesh is refined uniformly by a factor of two in each of the space dimensions and in the time dimension. The characteristic mesh numbers are provided in Table 1. We summarize the calculated errors and their experimental order of convergence (EOC) for the proposed space-time discretization in Table 2 and further illustrate them in Fig. 1. The numerical results confirm the expected third order rate of convergence established in Theorem 4.6 (cf. also Remark 4.7) for the discretization in the space-time domain with polynomial order $r=2$ and $p=2$, respectively, in the definition of the underlying finite element spaces. We note that the theoretically optimal order of convergence in time and space is obtained for the primal and the flux variable. Thus, the estimate (4.42) might be suboptimal with respect to the time discretization; cf. Remark 4.7. The estimate (4.43) is nicely confirmed by the presented numerical results. Further, we note that the optimal rate of convergence is obtained for the spatial discretization of the flux field in the norm of $\varvec{V}$. In this point the family of Raviart–Thomas pairs of mixed finite elements is superior to the family of Brezzi–Douglas–Marini pairs of mixed finite elements (cf. [17]) for that the optimal order of convergence of the flux variable can be obtained only in the norm of $\varvec{L}^2(\Omega )$.

**Table 1:** Space-time mesh with number of time subintervals *N*, global time discretization parameter $\tau _n$, number of cells $|\mathcal T_h|$, global space discretization parameter *h* and degrees of freedom $N_{ DoF }$ per degree of freedom in time

Level	*N*	$\tau _n$	$|\mathcal T_h|$	*h*	$N_{ DoF }$
0	10	1.000e$-$01	1	1.4142e$-$00	33
1	20	5.000e$-$02	4	7.0711e$-$01	120
2	40	2.500e$-$02	16	3.5355e$-$01	456
3	80	1.250e$-$02	64	1.7678e$-$01	1776
4	160	6.250e$-$03	256	8.8388e$-$02	7008
5	320	3.125e$-$03	1024	4.4194e$-$02	27,840

**Table 2:** Norm values and corresponding experimental orders of convergence in space-time for cGP(2)–MFEM(2) on the refinement levels as given in Table 1

Level	$\big \Vert e_u^{ cGP(2) } \big \Vert _{L^2(I; L^2(\Omega ))}$	EOC	$\big \Vert e_{{\varvec{q}}}^{ cGP(2) } \big \Vert _{L^2(I; \varvec{V})}$	EOC
0	4.0298e$-$02	–	8.2000e$-$01	–
1	1.1316e$-$02	1.83	2.2827e$-$01	1.84
2	1.4371e$-$03	2.98	2.8876e$-$02	2.98
3	1.8037e$-$04	2.99	3.6208e$-$03	3.00
4	2.2569e$-$05	3.00	4.5295e$-$04	3.00
5	2.8219e$-$06	3.00	5.6631e$-$05	3.00

### Distorted meshes

**Fig. 2:**
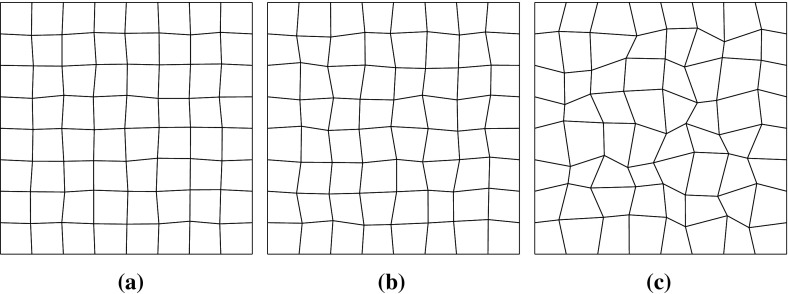
Distorted spatial meshes for $5\%$ (**a**), $10\%$ (**b**) and $25\%$ (**c**) random vertex movement for refinement level 3

In the second part of the numerical convergence studies we approximate the same analytical solution as before but we use spatial meshes with randomly distorted interior vertices. Precisely, in each of the computations we start on a coarse mesh and do uniform refinement steps by halvening the spatial mesh width. On the thus obtained finest mesh each of the interior vertices is distorted by a randomly chosen vector. The magnitude of the distortion vector is chosen randomly up to a given factor of relative length to the corresponding edge length. The characteristic numbers of the refinement levels are summarized in Table 3. The resulting distorted meshes are illustrated in Fig. 2 for the refinement level 3. The temporal mesh is chosen in the same way as in the first numerical experiment; cf. Table 1.

**Table 3:** Distorted spatial mesh: $h_\mathrm {max}$ largest cell diameter and $h_\mathrm {red}$ cell diameter reduction factor for 0%, 5%, 10% and 25% random vertex movement

Level	0%		5%		10%		25%	
	$h_\mathrm {max}$	$h_\mathrm {red}$	$h_\mathrm {max}$	$h_\mathrm {red}$	$h_\mathrm {max}$	$h_\mathrm {red}$	$h_\mathrm {max}$	$h_\mathrm {red}$
0	1.4142	–	1.4142	–	1.4142	–	1.4142	–
1	0.7071	2.00	0.7320	1.93	0.7570	1.87	0.8127	1.70
2	0.3536	2.00	0.3673	1.99	0.3814	1.98	0.4694	1.96
3	0.1768	2.00	0.1885	1.95	0.2002	1.91	0.2393	1.81
4	0.0884	2.00	0.0946	1.99	0.1008	1.99	0.1195	1.97
5	0.0442	2.00	0.0473	2.00	0.0504	2.00	0.0598	2.00

**Table 4:** Calculated errors and corresponding experimental order of convergence for $\big \Vert e_u^{ cGP(2) } \big \Vert _{L^2(I; L^2(\Omega ))}$ on distorted meshes given in Table 3

Level	5%	EOC	10%	EOC	25%	EOC
0	4.0298e$-$02	–	4.0298e$-$02	–	4.0298e$-$02	–
1	1.1353e$-$02	1.83	1.1463e$-$02	1.81	1.2155e$-$02	1.73
2	1.4381e$-$03	2.98	1.4783e$-$03	2.95	1.8690e$-$03	2.70
3	1.8290e$-$04	2.97	1.9117e$-$04	2.95	2.5232e$-$04	2.89
4	2.3024e$-$05	2.99	2.4402e$-$05	2.97	3.4463e$-$05	2.87
5	2.8787e$-$06	3.00	3.0483e$-$06	3.00	4.2870e$-$06	3.01

**Table 5:** Calculated errors and corresponding experimental order of convergence for $\big \Vert e_{{\varvec{q}}}^{ cGP(2) } \big \Vert _{L^2(I; \varvec{L}^2(\Omega ))}$ on distorted meshes given in Table 3

Level	5%	EOC	10%	EOC	25%	EOC
0	1.6206e$-$01	–	1.6206e$-$01	–	1.6206e$-$01	–
1	4.1287e$-$02	1.97	4.1460e$-$02	1.97	4.2889e$-$02	1.92
2	5.1846e$-$03	2.99	5.2156e$-$03	2.99	5.8069e$-$03	2.88
3	6.5995e$-$04	2.97	6.7916e$-$04	2.94	8.1938e$-$04	2.83
4	8.2527e$-$05	3.00	8.5013e$-$05	3.00	1.0344e$-$04	2.99
5	1.0310e$-$05	3.00	1.0628e$-$05	3.00	1.2982e$-$05	2.99

**Table 6:** Calculated errors and corresponding experimental order of convergence for $\big \Vert e_{{\varvec{q}}}^{ cGP(2) } \big \Vert _{L^2(I; \varvec{V})}$ on distorted meshes given in Table 3

Level	5%	EOC	10%	EOC	25%	EOC
0	8.2000e$-$01	–	8.2000e$-$01	–	8.2000e$-$01	–
1	2.2964e$-$01	1.84	2.3376e$-$01	1.81	2.6241e$-$01	1.64
2	3.0172e$-$02	2.93	3.5136e$-$02	2.73	7.5321e$-$02	1.80
3	3.9244e$-$03	2.94	4.7838e$-$03	2.88	1.0391e$-$02	2.86
4	6.0990e$-$04	2.69	9.6250e$-$04	2.31	2.7408e$-$03	1.92
5	1.1175e$-$04	2.45	2.0797e$-$04	2.21	6.4287e$-$04	2.09

**Fig. 3:**
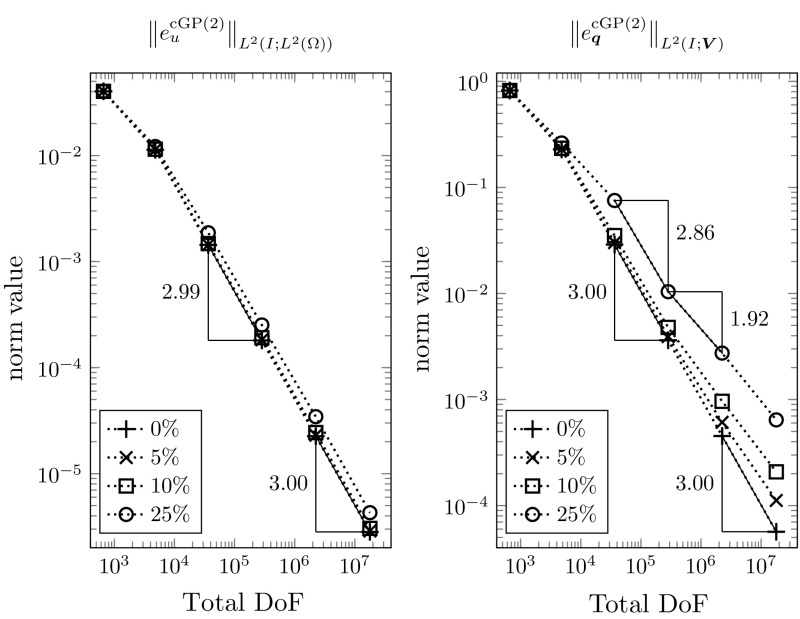
Calculated errors and corresponding experimental order of convergence on distorted meshes given in Table 3

We summarize the calculated errors and the corresponding experimental order of convergence (EOC) for the proposed space-time discretization on the distorted spatial meshes in Table 4 for the scalar-valued primal variable and in Tables 5 and 6 for the vector-valued flux variable and further illustrate them in Fig. 3. Tables 5 and 6 differ by the norms in that the errors of the flux approximation are measured. In Tables 4 and 5 the expected order of convergence in space and time, for the primal variable measured in the norm of $L^2(I;L^2(\Omega ))$ and for the flux variable measured in the norm of $L^2(I; \varvec{L}^2(\Omega ))$, is largely confirmed even for the strongly perturbed meshes with a distortion factor of 25%. This nicely demonstrates the robustness of the numerical scheme. Solely in Table 6 a slight reduction of the experimental order of convergence is observed depending on the degree of mesh perturbation. On the randomly distorted meshes the quasi uniformity condition, that is typically assumed about the finite element meshes in the numerical analyses, deteriorates successively. We conjecture that this impacts the convergence behavior in the stronger $L^2(I; \varvec{V} )$ norm more severely than in the $L^2(I; \varvec{L}^2(\Omega ))$ norm. The higher sensitivity of the derivatives in the $L^2(I; \varvec{V} )$ norm with respect to the mesh perturbations seems to be quite natural. Nevertheless, we note that even though a strong random mesh perturbation is applied, a robust convergence behavior is still ensured and optimal order of convergence in the $L^2(I; \varvec{L}^2(\Omega ))$ norm is obtained. Finally, we note that the space-time convergence studies on the distorted spatial meshes were done with exactly the same numerical solver settings as for the above-given studies on uniform meshes.

## Conclusions

In this work a numerical analysis of a family of variational space approximation schemes that combine continuous finite elements in time with the MFEM in space was presented for a parabolic prototype model of flow in porous media. The existence and uniqueness of the temporally semidiscrete and the fully discrete approximations were proved. Error estimates with explicit rates of convergence, including an optimal order error estimate, in natural norms of the scheme were established. The error estimates were illustrated and confirmed by numerical convergence studies. We believe that our analyses and techniques can be extended and applied to more sophisticated flow and transport processes in porous media or to incompressible viscous free flow. This will be our work for the future.

## A Supplementary proofs

In the sequel we introduce a variational semidiscretization in time of the weak formulation of the second order problem (2.6)–(2.8), i.e. without rewriting Eq. (2.6) as a first order system of equations as it is done in Sect. 2.3. Then we prove the existence and uniqueness of solutions to the resulting semidiscrete variational problem. This results is used to establish the existence of the semidiscrete approximation in mixed form defined by the variational problem (2.14), (2.15) in Sect. 3. Here, we present a different technique of proof than in [46] since one of the arguments that is used [46, Lemma 6.1] does not hold in the applied form from our point of view. Thereby, we aim to keep our work self-contained. Further, we summarize the proof of Theorem 3.10.

### A.1 Variational time discretization of the second order problem

In the following we use the notation that is introduced in Sects. 2.1 and 2.3, respectively. Moreover we use the splitting [cf. Eq. (3.12)]

$$\begin{aligned} u_\tau (t) = u_0 + u^0_\tau (t) \quad \text {with}\quad u^0_\tau \in \mathcal {X}_0^{r}(H^1_0(\Omega )). \end{aligned}$$

Further, we put

$$\begin{aligned} f_0 (t) = f(t) - A u_0 \end{aligned}$$

for $u_0 \in H^1_0(\Omega )$ such that by definition $Au_0 \in H^{-1}(\Omega )$, cf. Sect. 2.1. Under the additional regularity condition that

$$\begin{aligned} D(A)=H^2(\Omega )\cap H^1_0(\Omega ) \end{aligned}$$

it even holds that $f_0\in L^2(I ; L^2(\Omega ))$. The semidiscrete variational approximation of the system (2.6)–(2.8) is now defined by: *Find* $u_\tau ^0 \in X^r_0 (H^1_0(\Omega ))$ *such that*

A.1 $$\begin{aligned} \int _0^T \langle \partial _t u_\tau ^0, w_\tau \rangle \,\mathrm {d}t + \int _0^T a(u_\tau ^0,w_\tau ) \,\mathrm {d}t = \int _0^T \langle f_0, w_\tau \rangle \,\mathrm {d}t \end{aligned}$$

*for all* $$w_\tau \in Y^{r-1}(H^1_0(\Omega ))$$.

Firstly, we show the uniqueness of solutions to (A.1). In the sequel we denote by $\varphi _{n,j}=\varphi _{n,j}(t)$ for $j=0,\ldots ,r$ the Lagrange basis functions in $I_n=(t_{n-1},t_n]$ with respect to $r+1$ quadrature points $t_{n,l}$, $l=0,\ldots ,r$. Here, we choose the Gauss–Lobatto quadrature rule that is exact for polynomials of maximum degree $2r-1$. In particular, for the quadrature nodes in $\overline{I_n}$ it holds that $t_{n,0}=t_{n-1}$ and $t_{n,r}=t_n$. Then, any function $u_\tau ^0 \in X^r_0 (H^1_0(\Omega ))$ and its time derivative admit the representation

A.2 $$\begin{aligned} u_\tau ^0 (t) = \sum _{j=0}^r U_n^j \varphi _{n,j}(t),\quad \partial _t u_\tau ^0 (t) = \sum _{j=0}^r U_n^j \varphi _{n,j}^\prime (t) \end{aligned}$$

for all $t\in I_n$ with coefficient functions $U_n^j \in H^1_0(\Omega )$ for $j=0,\ldots ,r$.

#### Theorem A.1

[Uniqueness of solutions to (A.1)] Let the assumptions of Sect. 2.2 about $\Omega , u_0$ and *f* be satisfied. Then the solution $u_\tau ^0\in \mathcal {X}^{r}_0(H^1_0(\Omega ))$ of the semidiscrete problem (A.1) is unique.

#### Proof

Let $u_{\tau ,1}^{0}$, $u_{\tau ,2}^{0}\in \mathcal {X}^r_0{(H^1_0(\Omega ))}$ denote two solutions of the semidiscrete variational problem (A.1). We put $u_\tau ^0(t):= u_{\tau ,1}^{0} - u_{\tau ,2}^{0}$. We choose the test function $w_\tau := A^{-1} \partial _t u_\tau ^0 + \mu \partial _t u_\tau ^0$ for some fixed parameter $\mu \ge 0$. By means of (A.2), it holds that $w_\tau \in \mathcal {Y}^{r-1}{(H^1_0(\Omega ))}$. For this choice of $w_\tau$ it follows that

A.3 $$\begin{aligned} I:= \int _0^T \langle \partial _t u_\tau ^0(t), A^{-1} \partial _t u_\tau ^0 + \mu \partial _t u_\tau ^0 \rangle \,\mathrm {d}t +\int _0^T \langle A u_\tau ^0(t), A^{-1} \partial _t u_\tau ^0 + \mu \partial _t u_\tau ^0 \rangle \,\mathrm {d}t = 0 . \end{aligned}$$

By the symmetry of $a(\cdot ,\cdot )$ we have that $a(u_\tau ^0,\partial _t u_\tau ^0) = \frac{1}{2\,}\frac{d}{dt} a(u_\tau ^0 , u_\tau ^0 )$. Further, we have that $\langle u_\tau ^0, \partial _t u_\tau ^0 \rangle = \frac{1}{2\,}\frac{d}{dt} \Vert u_\tau ^0 \Vert ^2_{L^2(\Omega )}$. Recalling (2.3)–(2.5) and noting that $u_\tau ^0(0)=0$ and $\partial _t u_\tau ^0\in \mathcal {Y}^{r-1}{(H^1_0(\Omega ))}$, it follows from (A.3) that

$$\begin{aligned} 0&= I = \int _0^T \langle \partial _t u_\tau ^0(t), A^{-1} \partial _t u_\tau ^0 \rangle \,\mathrm {d}t + \int _0^T \langle \partial _t u_\tau ^0(t), \mu \partial _t u_\tau ^0 \rangle \,\mathrm {d}t\\&\quad + \int _0^T \langle A u_\tau (t), A^{-1} \partial _t u_\tau ^0 \rangle \,\mathrm {d}t + \int _0^T \langle A u_\tau ^0 (t), \mu \partial _t u_\tau ^0 \rangle \,\mathrm {d}t\\&\ge c \int _0^T \Vert \partial _t u_\tau ^0 \Vert _{H^{-1}( \Omega )}^2 \,\mathrm {d}t + \mu \int _0^T \Vert \partial _t u_\tau ^0 \Vert _{L^2(\Omega )}^2 \,\mathrm {d}t \\&\quad + \int _0^T \frac{1}{2}\frac{d}{dt} \Vert u_\tau ^0 \Vert _{L^2(\Omega )}^2 \,\mathrm {d}t + \mu \int _0^T \frac{1}{2}\frac{d}{dt} a(u_\tau ^0,u_\tau ^0) \,\mathrm {d}t \\&\ge c \int _0^T \Vert \partial _t u_\tau ^0 \Vert _{H^{-1}(\Omega )}^2 \,\mathrm {d}t + \mu \int _0^T \Vert \partial _t u_\tau ^0 \Vert _{L^2(\Omega )}^2 \,\mathrm {d}t\\&\quad + \frac{1}{2}\, \Vert u_\tau ^0 (T)\Vert _{L^2(\Omega )}^2 + \frac{\alpha \mu }{2} \Vert u_\tau ^0(T)\Vert ^2_{H^1_0(\Omega )}. \end{aligned}$$

This implies that $u_\tau ^0 =0$ and, consequently, that $u_{\tau ,1}^{0} = u_{\tau ,2}^{0}$. The uniqueness of solutions to (A.1) is thus established. $\square$

We remark that testing Eq. (A.1) with $v_\tau = A^{-1}\partial _t u_\tau ^0$ or $v_\tau = \partial _\tau u_\tau ^0$ would already be sufficient for proving the uniqueness result. Further, the symmetry of $a(\cdot ,\cdot )$ is essential in the previous proof. A generalization of the arguments to problems with nonsymmetric bilinear forms, for instance to convection–diffusion equations, still remains an open problem.

The existence of a solution to the semidiscrete problem (A.1) follows from the uniqueness of the solutions. Using the eigenspaces of *A*, problem (A.1) can be reduced to a set of finite dimensional problems, for each of which obviously uniqueness implies existence. For this we recall the following result from [26, Appendix D.6].

#### Lemma A.2

Let *H* be a separable Hilbert space, and suppose that $S: H\mapsto H$ is a compact and symmetric operator. Then there exists a countable orthonormal basis of *H* consisting of eigenfunctions of *S*.

#### Theorem A.3

[Existence of solutions to (A.1)] Let the assumptions of Sect. 2.2 about $\Omega , u_0$ and *f* as the be satisfied. Then the semidiscrete problem (A.1) admits a solution $u_\tau ^0\in \mathcal {X}^{r}_0(H^1_0(\Omega ))$.

#### Proof

The operator $S:=A^{-1}: L^2(\Omega )\mapsto L^2(\Omega )$ with *A* being defined in (2.1) is a bounded, linear compact operator mapping $L^2(\Omega )$ into itself. By means of Lemma A.2 there exists a set of appropriately scaled eigenfunctions $\{w_k\}_{k=1}^\infty \subset L^2(\Omega )$ with $w_k\in H^1_0(\Omega )$ such that $\{w_k\}_{k=1}^\infty$ is an orthogonal basis of $H^1_0(\Omega )$ and an orthonormal basis of $L^2(\Omega )$.

In terms of these eigenfunctions $\{w_k\}_{k=1}^\infty \subset H^1_0(\Omega )$ the solution $u_\tau ^0$ of problem (A.1) can be represented as

$$\begin{aligned} u_\tau (x,t) = \sum _{j=0}^r U_{n}^{(j)}(x) \varphi _{n}^{(j)}(t) = \sum _{j=0}^r\sum _{k=1}^\infty d_{n,k}^{(j)} w_k(x) \varphi _{n}^{(j)}(t), \quad \text{ for } t\in \overline{I}_{n} \end{aligned}$$

with coefficients $d_{n,k}^{(j)}\in \mathbb {R}$ for $k=1,\ldots ,\infty$ and each $j=0,\ldots ,r$ and $n=0,\ldots , N$. We choose test functions $v_\tau \in \mathcal {Y}^{r-1}(H^1_0(\Omega ))$ being defined by

$$\begin{aligned} v_\tau = \left\{ \begin{array}{ll} w_k \psi _{n}^{(i)}, &{}\quad \text{ for } t \in \overline{I}_n,\\ 0 , &{}\quad \text{ for } t \in I \backslash \overline{I}_n \end{array} \right. \end{aligned}$$

for $i=1,\ldots , r$, $k=1,\ldots , \infty$ and $n=1,\ldots ,N$. Then, for each $k=1,\ldots , \infty$, we get the finite dimensional problem

A.4 $$\begin{aligned} \begin{aligned}&\sum _{j=0}^r d_{n,k}^{(j)} \underbrace{\int _{I_n} d_t \varphi _{n}^{(j)}(t) \psi _{n}^{(i)}(t) \,\mathrm {d}t}_{:=\alpha _{ij}} +\sum _{j=0}^r d_{n,k}^{(j)} \underbrace{a(w_k,w_k)}_{:=\gamma _k} \underbrace{\int _{I_n} \varphi _{n}^{(j)}(t) \psi _{n}^{(i)}(t) \,\mathrm {d}t}_{=:\beta _{ij}} \\&\quad = \underbrace{\int _0^T \langle f_0(t),w_k \rangle \psi _n^{(i)}(t) \,\mathrm {d}t}_{=: b_{k,i}} \end{aligned} \end{aligned}$$

for $i=1,\ldots r$ and $n=1,\ldots ,N$. Due to the continuity of functions $u_\tau \in \mathcal {X}^r_0{(H^1_0(\Omega ))}$ and the choice of the Gauss–Lobatto quadrature rule it holds that

$$\begin{aligned} U_n^{(0)} = U_{n-1}^{(r)} \qquad \text{ or } \qquad d_{n,k}^{(0)} = d_{n-1,k}^{(r)}, \end{aligned}$$

respectively. Therefore, we recast the finite dimensional problem (A.4) as

A.5 $$\begin{aligned} \sum _{j=1}^r \left( \alpha _{ij} + \gamma _k \beta _{ij}\right) d_{n,k}^{(j)} = b_{k,i} + \left( \alpha _{i0} + \gamma _k \beta _{i0}\right) d_{n,k}^{(0)} \end{aligned}$$

for $i=1,\ldots r$ and each $k=1,\ldots ,\infty$. For the finite dimensional problem (A.5) the uniqueness of the solution established in Theorem A.1 then implies the existence of a solution. $\square$

Similarly to Corollary 3.4, the existence and uniqueness of the semidiscrete solution implies that an inf–sup stability condition in the underlying space-time framework is satisfied [24, p. 85, Thm. 2.6].

#### Corollary A.4

Let the assumptions of Sect. 2.2 about $\Omega , u_0, \varvec{D}$ and *f* be satisfied. Let $u_\tau ^0\in \mathcal {X}^{r}_0(H^1_0(\Omega ))$ be the unique solution of the semidiscrete problem (A.1) according to Theorems A.1 and A.3. Then, there exists a constant $c > 0$ such that

$$\begin{aligned} \inf _{u_\tau \in \mathcal {X}^r_0{(H^1_0(\Omega ))}\backslash \{0\}} \sup _{v_\tau \in \mathcal {Y}^{r-1}{(H^1_0(\Omega ))}\backslash \{0\}} \dfrac{B(u_\tau ,v_\tau )}{\Vert u_\tau \Vert _{\mathcal {X}}\Vert v_\tau \Vert _{\mathcal {Y}}} \ge c > 0 \end{aligned}$$

with

A.6 $$\begin{aligned} B(u_\tau ,v_\tau )&= \int _0^T \langle \partial _t u_\tau + A u_\tau ,v_\tau \rangle \,\mathrm {d}t, \nonumber \\ \Vert u_\tau \Vert _{\mathcal {X}}&= \left( \Vert u_\tau \Vert ^2_{L^2(I,H^1_0(\Omega ))} + \Vert \partial _t u_\tau \Vert ^2_{L^2(I,H^{-1}(\Omega ))}\right) ^{1/2}, \nonumber \\ \Vert v_\tau \Vert _{\mathcal {Y}}&= \Vert u_\tau \Vert _{L^2(I,H^1_0(\Omega ))}. \end{aligned}$$

#### Remark A.5

By the arguments of [46, Thm. 6.2] the inf–sup stability condition implies an error estimate for the semidiscretization (A.1) where the error is measured in the corresponding natural norm (A.6) of the scheme.

### A.2 Proof of Theorem 3.10

#### Proof

We let $w_\tau := I_\tau u - u_\tau$, $\varvec{v}_\tau = \varvec{J}_\tau \varvec{q} - \varvec{q}_\tau$ with the interpolation operators $I_\tau$ and $\varvec{J}_\tau$ of Sect. 3.2. Using the inf–sup stability condition along with problem (2.10), (2.11) and the non-exact semidiscrete problem (3.39), (3.40), applying the inequality of Cauchy–Schwarz and the continuity (2.13) of $a_\tau (\cdot ,\cdot )$ we conclude that

By means of the approximation properties (3.16) to (3.18) we then get that

$$\begin{aligned} \Vert \{w_\tau ,\varvec{v}_\tau \} \Vert _{\mathcal {W}}\le & {} c \Bigg (\sum _{n=1}^N \tau _n^{2r} \Big \{\Vert \partial _t^{r+1} u\Vert ^2_{L^2(I_n;W)} \\&+\, \tau _n^2 \Vert \partial _t^{r+1} \varvec{q} \Vert ^2_{L^2(I_n;\varvec{V})} + \tau _n^2 \Vert \partial _t^{r+1} f \Vert ^2_{L^2(I_n;W)}\Big \}\Bigg )^{1/2}. \end{aligned}$$

Combining this estimate with the triangle inequality yields the assertion of Theorem 3.10. $\square$

## B Summary of notation

### Function spaces and norms

**Table:**

*I*, $\Omega$	Time and space domain, $I=(0,T]$
$L^2(\Omega )$, $H^p(\Omega )$, $H_0^1(\Omega )$, $W^{1,\infty }(\Omega )$	Standard Sobolev spaces
$H^{-1}(\Omega )$	Dual space of $H_0^1(\Omega )$
$\Vert \cdot \Vert$, $\Vert \cdot \Vert _p$, $\langle \cdot , \cdot \rangle$	Norms in $L^2(\Omega )$, $H^p(\Omega )$, inner product in $L^2(\Omega )$
*W*, $\varvec{V}$	$W=L^2(\Omega )$, $\varvec{V} = \varvec{H}(\text {div};\Omega )$
$\Vert \cdot \Vert _{\varvec{V}}$	$\Vert \cdot \Vert _{\varvec{V}} = (\Vert \cdot \Vert ^2+ \Vert \nabla \cdot (\cdot ) \Vert ^2)^{1/2}$
$C(\overline{I};X)$, $L^2(I;X)$, $H^1(I;X)$	Bogner spaces with values in the Banach space *X*
$\varvec{D}$, $u_0$, *f*	Data of the problem
*u*, $\varvec{q}$	Solution and flux of the continuous problem
*a*(*u*, *v*)	Bilinear form $a(u,v)= \langle \varvec{D} u, v\rangle$

### Time discretization

**Table:**

$\mathcal {X}^{r}{(W)}$, $\mathcal {X}^{r}{(\varvec{V})}$	Trial spaces of semidiscretization in time
$\mathcal {X}_0^{r}{(W)}$	Trial space of semidiscretization with $u_\tau (0)=0$
$\mathcal {Y}^{r-1}{(W)}$, $\mathcal {Y}^{r}{(\varvec{V})}$	Test spaces of semidiscretization in time
$\mathbb {P}_r(J;\,X)$	$\mathbb {P}_r(J;\,X) = \{p(t) = \sum _{j=0}^{r}{\xi _n^j\, t^j} \mid \xi _n^j \in X\}$
$\mathcal {W}$, $\mathcal {V}$	$\mathcal {W} = X_0^r (W) \times X^r (\varvec{V})$, $\mathcal {V} = Y^{r-1} (W) \times Y^{r-1} (\varvec{V})$
$I_n$, $\tau _n$	Subinterval $I_n=(t_{n-1},t_n]$, $\tau _n = t_n-t_{n-1}$
$t_{n,0}$	$t_{n,0}=t_{n-1}$
$t_{n,1},\ldots , t_{n,r}$	*r*-point Gaussian quadrature nodes
$\varphi _{n,j}(t)$	Lagrange polynomial on $I_n$ w.r.t $t_{n,0}, \ldots , t_{n,r}$
$\psi _{n,i}(t)$	Lagrange polynomial on $I_n$ w.r.t $t_{n,1}, \ldots , t_{n,r}$
$u_\tau$	$u_\tau {}_{|\overline{I}_n} (t ) = \sum _{j=0}^r U_n^j\, \varphi _{n,j}(t)$
	Semidiscrete solution
$\varvec{q}_\tau$	$\varvec{q}_\tau {}_{|\overline{I}_n} (t ) = \sum _{j=0}^r \varvec{Q}_n^j \, \varphi _{n,j}(t)$
	Semidiscrete flux
$\hat{I}$	$\hat{I} = [0,1]$ reference interval
$\hat{t}_{1},\ldots , \hat{t}_{r}$	Gaussian quadrature nodes on $\hat{I}$
$\hat{\omega }_{1},\ldots , \hat{\omega }_{r}$	Gaussian quadrature weights on $\hat{I}$
$\hat{\varphi }_j(\hat{t})$	Transformed Lagrange polynomial on $\hat{I}$
$\hat{\alpha }_{ij}$, $\hat{\beta }_{ij}$	$\hat{\alpha }_{ij} = \hat{\omega }_i$, $\hat{\beta }_{ij} = \hat{\omega }_i \delta _{i,j}$ with Kronecker symbol $\delta _{i,j}$
	From transformation of time integrals to $\hat{I}$ and
	Application of quadrature on $\hat{I}$
$\Pi _r$	Temporal Lagrange interpolant
	W.r.t. to $t_{n,0},\ldots , t_{n,r}$
$I_\tau$	Temporal interpolation operator for variable *u*
$\varvec{J}_\tau$	Temporal interpolation operator for variable $\varvec{q}$
$a_{\tau }(\{\cdot ,\cdot \},\{\cdot ,\cdot \})$	Space-time bilinear form
$u^0(t)$, $u_\tau ^0(t)$	$u^0(t) = u(t)-u_0$, $u_\tau ^0(t) = u_\tau (t)-u_0$
*z*, $\varvec{p}$	Solution of dual problem
$$ I_0$$	Temporal interpolation operator for *z*
$\varvec{J}_0$	Temporal interpolation operator for $\varvec{p}$

### Space discretization and error analysis

**Table:**

$W_h$, $\varvec{V}_h$	Inf–sup stable pair of finite element spaces
	Raviart–Thomas(–Nédélec) elements
$\mathcal {X}^{r}{(W_h)}$, $\mathcal {X}^{r}{(\varvec{V}_h)}$	Trial spaces of space-time discretization
$\mathcal {Y}^{r-1}{(W_h)}$, $\mathcal {Y}^{r-1}{(\varvec{V}_h)}$	Test spaces of space-time discretization
$u_{\tau ,h}$	$u_{\tau ,h} (t)_{|I_n} = \sum _{j=0}^r U_{n,h}^{j} \varphi _{n,j}(t)$
	Fully discrete solution
$\varvec{q}_{\tau ,h}$	$\varvec{q}_{\tau ,h} (t)_{|I_n} = \sum _{j=0}^r \varvec{Q}_{n,h}^{j} \varphi _{n,j}(t)$
	Fully discrete flux
$P_h$, $\varvec{P}_h$	$L^2$ projection onto $W_h$ and $\varvec{V}_h$
$\varvec{\Pi }_h$	Projection onto $\varvec{V}_h$:
	$\langle \nabla \cdot (\varvec{\Pi }_h \varvec{v} - \varvec{v}) , w_h \rangle = 0$ for all $w_h\in W_h$
$E_{u}(t)$	$E_{u}(t) = u_\tau (t) - u_{\tau ,h} (t)$
	$$E_{u}(t)=\sum _{j=0}^r E_{u,n}^j \varphi _{n,j}(t) $$
	Error between semidiscrete and fully discrete
	Approximation of scalar variable
$\varvec{E}_{\varvec{q}}(t)$	$\varvec{E}_{\varvec{q}}(t) =\varvec{q}_\tau (t) - \varvec{q}_{\tau ,h}(t)$
	$$\varvec{E}_{\varvec{q}}(t) = \sum _{j=0}^r \varvec{E}_{\varvec{q},n}^j \varphi _{n,j}(t)$$
	Error between semidiscrete and fully discrete
	Approximation of flux variable
$E_{u,n}^i$	$E_{u,n}^i = E_u(t_{n,i})$: error in node $t_{n,i}$, $i=0,\ldots ,r$
$\varvec{E}_{\varvec{q},n}^i$	$\varvec{E}_{\varvec{q},n}^i = \varvec{E}_{\varvec{q}}(t_{n,i})$: error in node $t_{n,i}$, $i=0,\ldots ,r$
